# Biological signature of dietary patterns in older adults: findings from a population-based study

**DOI:** 10.1186/s12916-026-05202-2

**Published:** 2026-09-15

**Authors:** Rachel Ann DaCosta, Caterina Gregorio, Amaia Calderón-Larrañaga, Alice Margherita Ornago, Anja Mrhar, Rosario Ortolá, Claudia Fredolini, Davide Liborio Vetrano, Adrián Carballo-Casla

**Affiliations:** 1 Aging Research Center, Department of Neurobiology, Care Sciences and Society, Karolinska Institutet and Stockholm University, Stockholm, Sweden; 2 Stockholm Gerontology Research Center, Stockholm, Sweden; 3 Biotechnical Faculty, University of Ljubljana, Ljubljana, Slovenia; 4 Department of Preventive Medicine and Public Health, Universidad Autónoma de Madrid, Madrid, Spain; 5 Center for Networked Biomedical Research in Epidemiology and Public Health (CIBERESP), Madrid, Spain; 6 Department of Protein Science, KTH Royal Institute of Technology, SciLifeLab, Solna, Sweden; 7 Affinity Proteomics Unit, KTH Royal Institute of Technology, SciLifeLab, Solna, Sweden

**Keywords:** Nutrition, Eating patterns, Geroscience, Dietary inflammatory potential, Inflammaging, Age-related diseases, Healthy aging, Biological processes

## Abstract

**Background:**

Diet quality is a key modifiable factor influencing age-related health outcomes, yet its impact on the biological mechanisms involved in the aging process is not fully understood. This study aimed to identify the blood-based biomarkers most strongly associated with adherence to diverse dietary patterns and to describe the pathophysiological domains they belong to.

**Methods:**

We analysed data from 1,769 participants aged ≥ 60 years from the population-based Swedish National Study on Aging and Care in Kungsholmen. Dietary intake was assessed using validated food frequency questionnaires. Diet quality was evaluated using adherence to the Alternative Mediterranean Diet (AMED), the Alternative Healthy Eating Index (AHEI), and the reverse-transformed Empirical Dietary Inflammatory Index (rEDII). Fifty-four blood biomarkers spanning metabolic, inflammatory, vascular, and neurodegenerative domains were analysed. Cross-sectional associations between adherence to the dietary patterns and standardized biomarker levels were examined using quantile regression, adjusted for sociodemographic and lifestyle factors.

**Results:**

After multiple comparison correction, at the median level of each biomarker distribution, higher adherence (1 standard deviation increment) to the AMED was directly associated with folic acid levels [beta coefficient (95% confidence interval) = 0.066 (0.033, 0.099)] and inversely with glial fibrillary acidic protein [-0.018 (-0.030, -0.006)]. Higher AHEI adherence showed direct association with folic acid levels [0.068 (0.035,0.100)] and inverse with growth differentiation factor 15 levels [-0.063 (-0.098, -0.029)]. Higher adherence to the rEDII was inversely associated with leptin [-0.094 (-0.139, -0.049)], insulin [-0.086 (-0.130, -0.043)], growth differentiation factor 15 [-0.064 (-0.100, -0.027)], alkaline phosphatase [-0.078 (-0.122, -0.033)], tumour necrosis factor receptor superfamily member 1B [-0.047 (-0.076, -0.019)], and leukocytes [-0.058 (-0.092, -0.024)]. Associations remained consistent across multiple sensitivity analyses.

**Conclusions:**

Higher diet quality is associated with favourable levels of blood-based biomarkers in older adults, with distinct associations within the metabolic and inflammatory domains, supporting the role of diet in promoting healthier aging.

**Supplementary Information:**

The online version contains supplementary material available at https://doi.org/10.1186/s12916-026-05202-2.

## Background

Rising global life expectancy reflects major advances in healthcare and disease management. However, a longer lifespan does not necessarily equate to a longer health span, that is, the years lived free from chronic disease and disability [1]. Advanced age remains the primary risk factor for multiple chronic, non-communicable diseases, including cardiovascular disease, type 2 diabetes, neurodegenerative disorders, and cancer, which are major contributors to mortality, low quality of life, and rising healthcare costs [2]. Consequently, identifying modifiable risk factors that promote healthy aging, preserve functional capacity, and reduce disease burden and related expenses is warranted [3].

Nutrition is a well-established, modifiable risk factor that influences age-related health outcomes [4]. Traditionally, nutritional research focused on isolated nutrients or individual foods. However, this reductionist approach fails to capture the complexity of real-world diets and synergistic interactions between dietary components [5]. In response, contemporary studies have shifted toward dietary pattern analysis, using composite indices that better reflect overall diet quality [6]. Multiple systematic reviews and meta-analyses demonstrate that adherence to high-quality dietary patterns like the Mediterranean diet, rich in fruits, vegetables, legumes, whole grains, fish and nuts, and low in red and processed meat, is associated with reduced risks of cardiovascular diseases, physical function decline, and cognitive impairment in older adults [7–9]. In parallel, observational studies have shown that higher adherence to other healthy dietary patterns, such as the Alternative Healthy Eating Index, which emphasizes fruits, vegetables, whole grains, legumes, nuts, and fish, and discourages sugary drinks and nutrients found in processed foods, is linked to a lower risk of multimorbidity, frailty, and all-cause mortality in aging populations [10–12]. In contrast, higher adherence to proinflammatory dietary patterns characterized by greater consumption of refined grains, sweetened beverages, red and processed meats, and unhealthy fats has been linked to increased risks of frailty, cognitive decline, and chronic disease burden in older adults [13, 14].

Despite these epidemiological associations, the biological processes through which adherence to dietary patterns influences aging remain unclear [15]. A major limitation in aging research is the reliance on chronological age, which fails to capture the considerable variability in health status among individuals of the same age [16]. To better capture this variability, researchers are increasingly using markers of biological age, a plethora of integrative measures based on molecular, cellular, and physiological biomarkers, that more accurately reflect an individual’s functional capacity and health status [17]. These biomarkers provide objective insight into multiple biological processes and, when interpreted together, could form a signature that offers a more comprehensive and sensitive measure of the underlying aging process, integrating the complex, multifactorial nature of aging [18].

Given the distinct pathophysiological changes accompanying aging and the well-established role of nutrition in modifying age-related outcomes, it is essential to understand how adherence to dietary patterns influences these very processes, which cannot be fully captured by chronological age alone [3, 4]. These insights may help reveal whether certain diets are associated with healthier aging or increased risk of age-related diseases and inform the development of precision nutrition strategies for older adults [4]. Although previous research has explored associations between dietary patterns and blood biomarkers, studies specifically targeting older populations remain limited [19–21]. Moreover, most existing studies in older adults have investigated dietary patterns in relation to individual biomarkers, rather than assessing broad biomarker panels that more comprehensively capture the complex and multifactorial pathophysiological processes relevant to aging [22, 23]. This study addresses that gap by examining the associations of three dietary patterns consistently linked with health outcomes in older adults: the Alternative Mediterranean Diet (AMED), the Alternative Healthy Eating Index (AHEI), and the Empirical Dietary Inflammatory Index (EDII) with a panel of blood-based biomarkers spanning key aging-relevant pathophysiological domains (i.e., metabolic, inflammatory, vascular, and neurodegenerative), aiming to identify those biomarkers most associated with dietary pattern adherence in this population [8–14].

## Methods

### Study design and population

This cross-sectional study used baseline data from the Swedish National Study on Aging and Care in Kungsholmen (SNAC-K), an ongoing longitudinal community-based cohort study designed to investigate aging processes and identify preventive strategies to enhance health and care in older adults [24]. Between 2001 and 2004 (baseline), an age-stratified random sample of 5,111 community-dwelling and institutionalized adults aged 60 years and older residing in central Stockholm were invited (via letters) to participate in the study. A total of 4,590 individuals were eligible, of whom 1,227 refused and 3,363 agreed to participate and were enrolled (participation rate 73%). Follow-up assessments have been conducted every 6 years for participants younger than 78 years, and every 3 years thereafter, as described elsewhere [24]. At each study visit, participants underwent thorough clinical examinations, completed questionnaires (including food frequency questionnaires [FFQs]), and were interviewed by physicians, trained nurses, and psychologists. This study uses baseline (wave1) dietary and blood-based biomarker data only, as comprehensive biomarker information is not available at subsequent follow-ups.

From the initial cohort (*n* = 3,363), we excluded individuals with missing blood sample for analysis (*n* = 834), missing at least one blood-based biomarker measurement (*n* = 328), missing or inadequate dietary data (i.e., > 50% of missing answers in the FFQs) (*n* = 427), and those missing data on any sociodemographic variable (*n* = 5). Due to the larger level of missingness (*n* = 150), participants with missing data on any lifestyle-related potential confounders were retained in analyses and assigned a separate missing category indicator. After exclusions, the final analytical sample comprised 1,769 older adults (Additional file 1: Fig. S1) .

### Variables

#### Dietary patterns

Baseline dietary intake was assessed using a validated 98-item FFQ [25]. Participants reported their habitual food and beverage consumption frequency over the previous year on a nine-point scale, ranging from “never or less than once per year” to “more than four times per day”. Portion sizes were estimated using standardized colour photographs, and daily energy and nutrient intake were calculated using the Swedish National Food Agency’s food composition Table [26]. Adherence to three validated dietary patterns—the AMED [27], the AHEI [28], and the EDII [29]—was evaluated through analysis of food groups, specific foods, and individual nutrients. These patterns were selected to enable clear comparisons of findings both across prior research—given their consistent associations with health outcomes in older adults—and within this study, to test the hypothesis that despite capturing conceptually distinct yet complementary dimensions of dietary intake, they could show comparable associations with biomarker outcomes. These dimensions reflect adherence to a Mediterranean-style diet (AMED), and an index of overall diet quality (AHEI), both constructed in relation to chronic disease risk, alongside a measure of dietary inflammatory potential (EDII) derived empirically from inflammatory biomarker data [9–14]. Dietary components’ intake and scoring for each dietary pattern, including recommended intake thresholds for the AHEI and inflammatory effect scores for the EDII, are provided in Additional file 1: Tables S1-S3.

The AMED score was calculated to assess adherence to a Mediterranean dietary pattern developed by Trichopoulou et al., based on an adapted version of the original score for a non-Greek population [27]. It also includes several modifications based on scientific evidence linking foods, nutrients, and chronic disease risk [30]. Participants received 1 point each for consumption at or above the study population median intake for presumably beneficial components (vegetables, legumes, fruits, nuts, whole grains, fish, and the monounsaturated-to-saturated fat ratio) and 1 point for consumption below the median for detrimental components (red meat/poultry, dairy). Moderate alcohol intake was also scored positively. Scores ranged from 0 to 9, with higher scores indicating greater adherence to the Mediterranean diet (Additional file 1: Table S1).

The AHEI, developed by Chiuve et al., assesses diet quality based on foods linked to chronic disease risk [28]. Eleven food/nutrient groups were scored: presumably beneficial components (vegetables, fruits, nuts and legumes, whole grains, polyunsaturated fats, long-chain omega-3 fats, and moderate alcohol intake) and detrimental components (red/processed meat, sugar-sweetened beverages, trans fat, and sodium). Each component contributes 0 to 10 points, based on predefined recommended intake levels and established scoring criteria. Total scores in our study ranged from 29.9 to 91.6, with higher scores reflecting a healthier diet (Additional file 1: Table S2).

EDII scores were calculated according to Tabung et al., who linked the consumption of 18 food groups with three inflammatory biomarkers: C-reactive protein, interleukin-6, and tumour necrosis factor α receptor 2 [29]. Unlike the AMED and AHEI, the EDII is empirically derived, with specific focus on dietary inflammatory potential as opposed to regional dietary habits or overall chronic disease risk reduction. The apparently pro-inflammatory components included processed meat, red meat, organ meat, non-dark-meat fish, non-leafy green vegetables (excluding dark yellow vegetables and tomatoes), refined grains, high-energy beverages, low-energy beverages, and tomatoes. Anti-inflammatory components comprised dark yellow vegetables, leafy greens, snacks, fruit juice, pizza, tea, coffee, beer, and wine. Each participant’s mean daily food group consumption was multiplied by its inflammatory effect score (ranging from − 1175 to 252). Weighted scores were divided by 100 and reverse-transformed for interpretability, so that higher values represent lower dietary inflammatory potential. Total scores in our study ranged from − 26.9 to 11.0 (Additional file 1: Table S3).

#### Blood-based biomarkers

A standard blood draw was performed at baseline as part of the SNAC-K protocol. Venous blood samples were obtained via peripheral venipuncture, with no requirement for fasting before collection. Laboratory assessments were performed at each wave at the haematological and biochemical laboratory at Karolinska Hospital, according to standardized institutional procedures within 12 hours after collection. The routinely performed measurements included haemoglobin, total cholesterol, albumin, alkaline phosphatase, calcium, creatinine, folic acid, gamma-glutamyl transferase, haemoglobin A1c, leucocytes, thyroid-stimulating hormone, thyroxine, and vitamin B12. Following centrifugation, serum aliquots were prepared and stored at -80 °C in cryogenic vials at the Karolinska Institutet Biobank for future analyses.

In this study, we analysed data on the 54 blood-based biomarkers available in the SNAC-K: the 13 routine laboratory markers outlined above and 41 newly measured markers, the latter being selected based on their relevance to four key pathophysiological processes implicated in aging: metabolic, inflammatory, vascular, and neurodegenerative. This selection was informed by a comprehensive review of the literature and expert consensus [31]. The newly measured biomarkers were quantified using multiple laboratory techniques presented in Additional file 1: Table S4. Serum protein biomarkers were quantified at the Affinity Proteomics-Stockholm unit (SciLifeLab, Solna, Sweden) using bead-based multiplexed assays on either a Luminex system or Single Molecule Array (Quanterix, Simoa) platform. For inflammatory and vascular disease-related biomarkers, a custom-designed Magnetic Luminex Assay (Human Premixed Multi-Analyte Kit, Luminex Corporation, Austin, TX, USA) was employed. Samples were diluted according to the manufacturer’s protocol, processed in a 384-well plate format following a validated standard operating procedure, and analyzed on a FlexMap 3D system (Luminex). Median Fluorescence Intensity values, obtained via the xPONENT software (Luminex), served as measures of relative protein quantification for both samples and standards. Subsequent data processing, including curve fitting, concentration extrapolation, and graphical representation, was conducted using Belysa™ Immunoassay Curve Fitting Software (Millipore), a specialized tool for secondary analysis of Luminex-derived data. Standard curves were generated using a five-parameter logistic regression model to ensure accurate quantification.

Neurofilament light chain and glial fibrillary acidic protein were assayed using a Simoa Neuro 2-plex B Kit. Amyloid beta peptides (amyloid beta protein 40, amyloid beta protein 42) and total tau were measured using a Simoa Neuro 3-plex A Kit. Phosphorylated tau at threonine 181 and phosphorylated tau at threonine 217 were quantified using the Simoa pTau-181 Advantage V2 Kit and Simoa ALZpath p-tau217 Advantage PLUS, respectively. For each kit, 25 µL of sample was diluted 1:4, and the assays were performed based on the manufacturer’s instructions. The Quanterix instrument provided average enzyme per bead values for calibrators, controls, and samples. Curve-fitting, extrapolation of concentrations, and graphical representations were automatically performed with the Quanterix SR-X software using the calibrators and a four-parameter logistic curve fit.

Baseline levels of interleukins (IL-1β, IL-6, IL-8, IL-10, IL-12p70), interferon gamma, and tumour necrosis factor alpha were measured at Accelerator Laboratory Services, Quanterix Corp., in Billerica (MA, USA), using Simoa CorPlex Human Cytokine Panel 1 on the Quanterix^®^ SP-XTM imaging and analysis platform. Sources of missing biomarkers and corresponding data handling procedures are summarized in Additional file 1: Table S5.

#### Additional variables

Sociodemographic variables included were age, sex, education (elementary, high school, university), living arrangement (alone or not alone), and primary lifelong occupation (manual or non-manual labour). Lifestyle factors included smoking status (current, former, never smoker, or missing), physical activity level (inadequate, health-enhancing, fitness-enhancing, or missing, based on self-reported frequency and intensity) [32], total daily energy intake (kcal/day, estimated from the FFQs), and fasting status prior to the blood draw (fasting or non-fasting).

In ancillary analyses, we used data on body mass index (BMI, calculated as weight in kilograms divided by height in metres squared [kg/m²]) (< 18.5, 18.5 to < 25, 25 to < 30, ≥30), chronic disease burden, which was defined as the total number of chronic diseases at baseline, based on a clinically curated list developed by an international team of geriatricians, general practitioners, and epidemiologists [33], and number of daily medications (recorded during the physician interviews or from medical records for institutionalized participants). A directed acyclic graph describing the potential causal and confounding effects of diet quality on blood-based biomarkers is shown in Additional file 1: Fig. S2.

### Statistical analyses

#### Descriptive analyses

Descriptive characteristics of the study population were summarized using frequencies with percentages for the total sample, as well as means and standard deviations (SD) of the AMED, AHEI, and reverse-transformed EDII (rEDII) scores at the different levels of the socio-demographic, lifestyle, and ancillary characteristics. The statistical significance of the differences in characteristics across levels of adherence to the dietary patterns was assessed using analysis of variance.

#### Main analyses

Separate quantile regression models were used to examine the associations between adherence to dietary patterns (per 1-SD increment, to enable comparability across patterns) and concentrations of each biomarker (standardized to z-scores, to ensure consistent scaling). Models estimated the median (50th percentile) of each biomarker distribution conditional on each dietary pattern score and potential confounders. Both unadjusted and adjusted models were estimated, the latter accounting for age, sex, education, occupation, living arrangement, smoking status, physical activity, total energy intake, and fasting status. For each dietary pattern-biomarker pair, β coefficients with two-sided p-values were extracted. All p-values were corrected for multiple comparison testing across 162 tests (3 dietary patterns x 54 biomarkers) using the Benjamini-Hochberg procedure, with statistical significance defined as q < 0.10, and alternatively at a more stringent threshold of q < 0.05 [34]. Analyses were performed using Stata BE 18.0 with the *qreg* command [35]. Visualizations were created using the *ggplot2* R Statistical Package [36].

#### Sensitivity analyses

Additional quantile regression models were conducted at the 25th and 75th percentiles of each biomarker distribution to assess whether associations with dietary pattern adherence differed at lower or higher biomarker levels. Median quantile regression was also repeated: (1) using the standard panel of biomarkers available at wave 3 (i.e., six-year follow-up) to assess the consistency of cross-sectional findings; (2) restricting to participants with complete data on all potential confounders (complete case analysis); (3) restricting to participants with ≤ 4 chronic diseases at baseline to assess potential reverse causation; (4) additionally adjusting for the number of medications; (5) additionally adjusting for chronic disease burden; (6) with stabilized inverse probability weights to account for potential selection into the analytic sample; and (7) stratified by sex to explore whether associations differed in males and females (including multiplicative interaction testing).

Two separate causal mediation analyses were conducted using the *mediate* command in Stata [35], to explore whether BMI and chronic disease burden mediated the significant associations (median quantile regression q < 0.10) between dietary pattern adherence and biomarkers. Dietary pattern adherence (per 1-SD increment) was specified as the exposure, with BMI modelled as a continuous mediator (linear regression) and chronic disease burden as a count mediator (Poisson regression), adjusting for the same covariates as the primary analyses. Natural indirect, natural direct, and total effects were estimated along with the proportion mediated.

## Results

### Descriptive results

Characteristics of the study sample are shown in Table 1. The mean age at baseline was 70.5 years (SD = 9.31), and 60% of the participants were females. Participants with higher adherence to healthier dietary patterns were generally younger, more educated, more likely to cohabitate, lifelong non-manual workers, more physically active, had BMI values in the normal to overweight range (18.5 to < 30 kg/m²), had fewer chronic diseases, and were taking fewer medications. Participants excluded from the analyses tended to be older, less educated, more often lifelong manual workers, had more chronic diseases, and higher medication use compared to those included in the analyses (Additional file 1: Table S6).

**Table 1:** Adherence to the dietary patterns across categories of characteristics of the study population

Characteristics	*N* = 1769 *n* (%)	AMED Range (0 to 9) Mean (SD)	AHEI Range (29.9 to 91.6) Mean (SD)	rEDII Range (-26.9 to 11.0) Mean (SD)
Age (years)				
60 to < 65	560 (31.6)	4.55 (1.74) *	63.7 (9.41) *	0.35 (3.07) *
65 to < 70	360 (20.4)	4.36 (1.83)	62.2 (10.4)	-0.43 (3.10)
70 to < 80	525 (29.7)	4.37 (1.69)	61.7 (9.22)	-0.73 (2.82)
≥ 80	324 (18.3)	3.70 (1.59)	57.0 (9.74)	-1.25 (3.10)
Sex				
Male	707 (39.9)	4.36 (1.69)	59.7 (9.92) *	-0.41 (3.14)
Female	1062 (60.0)	4.26 (1.77)	62.8 (9.65)	-0.42 (3.01)
Living arrangement				
Alone	873 (49.3)	4.08 (1.76) *	60.7 (10.1) *	-0.59 (3.18) *
Not alone	896 (50.7)	4.51 (1.69)	62.4 (9.57)	-0.25 (2.94)
Previous occupation				
Manual worker	343 (19.4)	4.08 (1.67) *	59.5 (10.0) *	-1.21 (3.45) *
Non-manual worker	1426 (80.6)	4.35 (1.75)	62.1 (9.78)	-0.23 (2.93)
Education				
Elementary	229 (12.9)	3.95 (1.69) *	58.2 (10.2) *	-1.36 (3.21) *
High school	864 (48.9)	4.15 (1.71)	61.1 (9.38)	-0.64 (3.03)
University	676 (38.2)	4.61 (1.75)	63.3 (10.1)	0.18 (2.94)
Tobacco smoking				
Never	752 (43.1)	4.34 (1.71) *	61.9 (9.61) *	-0.70 (2.95) *
Former smoker	714 (40.9)	4.39 (1.78)	62.2 (9.58)	-0.21 (2.91)
Current smoker	278 (15.9)	3.96 (1.69)	59.2 (10.7)	0.00 (3.54)
Physical activity				
Inadequate	305 (18.3)	3.75 (1.70) *	58.3 (9.96) *	-0.54 (2.81) *
Health-enhancing	891 (53.6)	4.28 (1.70)	61.5 (9.67)	-0.51 (3.13)
Fitness-enhancing	465 (28.0)	4.78 (1.68)	64.4 (9.27)	0.35 (2.95)
Energy intake (kcal/day)				
< 1500	442 (25.0)	3.50 (1.56) *	62.2 (9.22) *	0.24 (2.21) *
1500 to < 2000	542 (30.6)	4.20 (1.68)	63.1 (9.49)	0.07 (2.49)
≥ 2000	785 (44.4)	4.81 (1.70)	60.1 (10.3)	-1.13 (3.64)
Fasting status				
Yes	59 (3.34)	4.42 (1.76)	62.7 (9.64)	-0.06 (0.28)
No	1687 (95.4)	4.29 (1.74)	61.5 (9.89)	-0.04 (0.30)
Body mass index (kg/m^2^)				
< 18.5	27 (1.54)	3.48 (1.78) *	55.2 (11.8) *	-1.22 (3.17) *
18.5 to < 25	761 (43.4)	4.25 (1.77)	61.5 (10.1)	-0.43 (3.22)
25 to < 30	727 (41.5)	4.41 (1.67)	62.3 (9.53)	-0.16 (2.79)
≥ 30	239 (13.6)	4.18 (1.38)	60.3 (9.76)	-1.02 (3.12)
Chronic diseases (number)				
0–1	292 (16.5)	4.63 (1.63) *	64.4 (9.38) *	0.50 (2.58) *
2–3	681 (38.5)	4.37 (1.76)	62.2 (10.0)	-0.25 (3.18)
4–5	476 (26.9)	4.21 (1.67)	60.3 (9.63)	-0.72 (31.0)
≥ 6	320 (18.1)	3.97 (1.83)	59.4 (9.72)	-1.17 (2.91)
Number of medications				
0–1	576 (32.6)	4.41 (1.68)	62.2 (9.69)	0.01 (2.92) *
2–4	670 (37.8)	4.29 (1.74)	61.6 (10.2)	-0.43 (3.09)
≥ 5	523 (29.5)	4.19 (1.80)	60.7 (9.64)	-0.87 (3.12)

AMED: Alternative Mediterranean Diet; AHEI: Alternative Healthy Eating Index; rEDII:reverse-transformed Empirical Dietary Inflammatory Score. *Indicates a statistically significant difference (p < 0.05) in adherence to a given dietary pattern (per 1-SD increment) across categories within each variable, tested using one-way analysis of variance. The number of participants with missing data was 25 for tobacco smoking, 108 for physical activity, 23 for fasting status and 15 for body mass index

### Main results

**Fig. 1:**
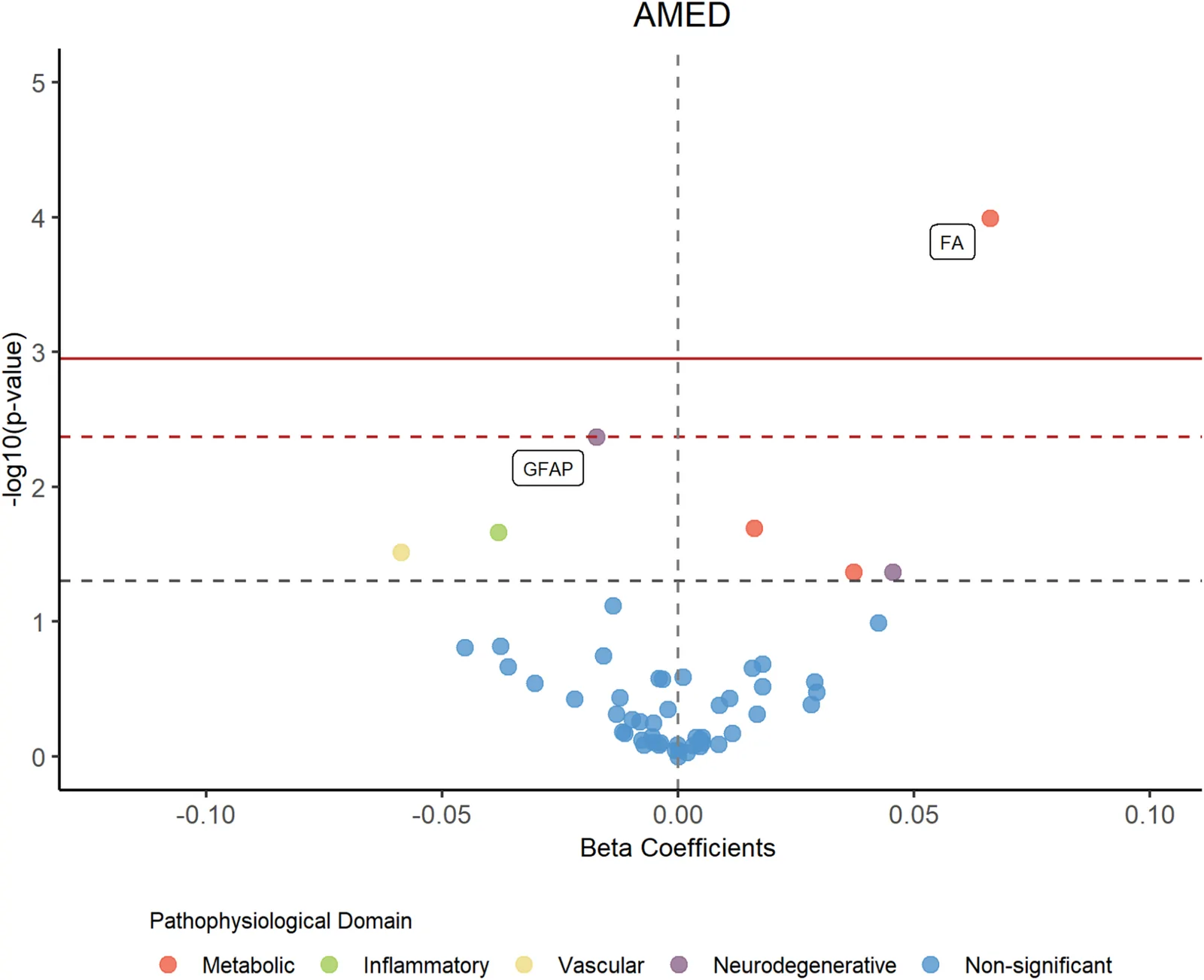
Volcano plots displaying the association between adherence to the Alternative Mediterranean Diet (AMED) (per 1-SD increment) and blood-based biomarkers at the median (50th percentile) of the biomarkers’ distributions. Beta coefficients are taken from separate quantile regression models (one for each dietary pattern-biomarker combination) adjusted for age, sex, education, occupation, living arrangement, physical activity, smoking status, energy intake, and fasting status. The absolute value of each coefficient represents the strength of the association between adherence to a given dietary pattern and levels of a blood-based biomarker (across different pathophysiological domains), while the sign indicates the direction (direct or inverse) of the relationship. The horizontal dashed grey line indicates the threshold for statistical significance (p < 0.05). The horizontal dashed red line indicates the Benjamini-Hochberg corrected threshold for statistical significance (q < 0.10) and the solid red line indicates a more stringent threshold (q < 0.05). AMED = Alternative Mediterranean Diet; FA = Folic acid; GFAP = Glial fibrillary acidic protein

**Fig. 2:**
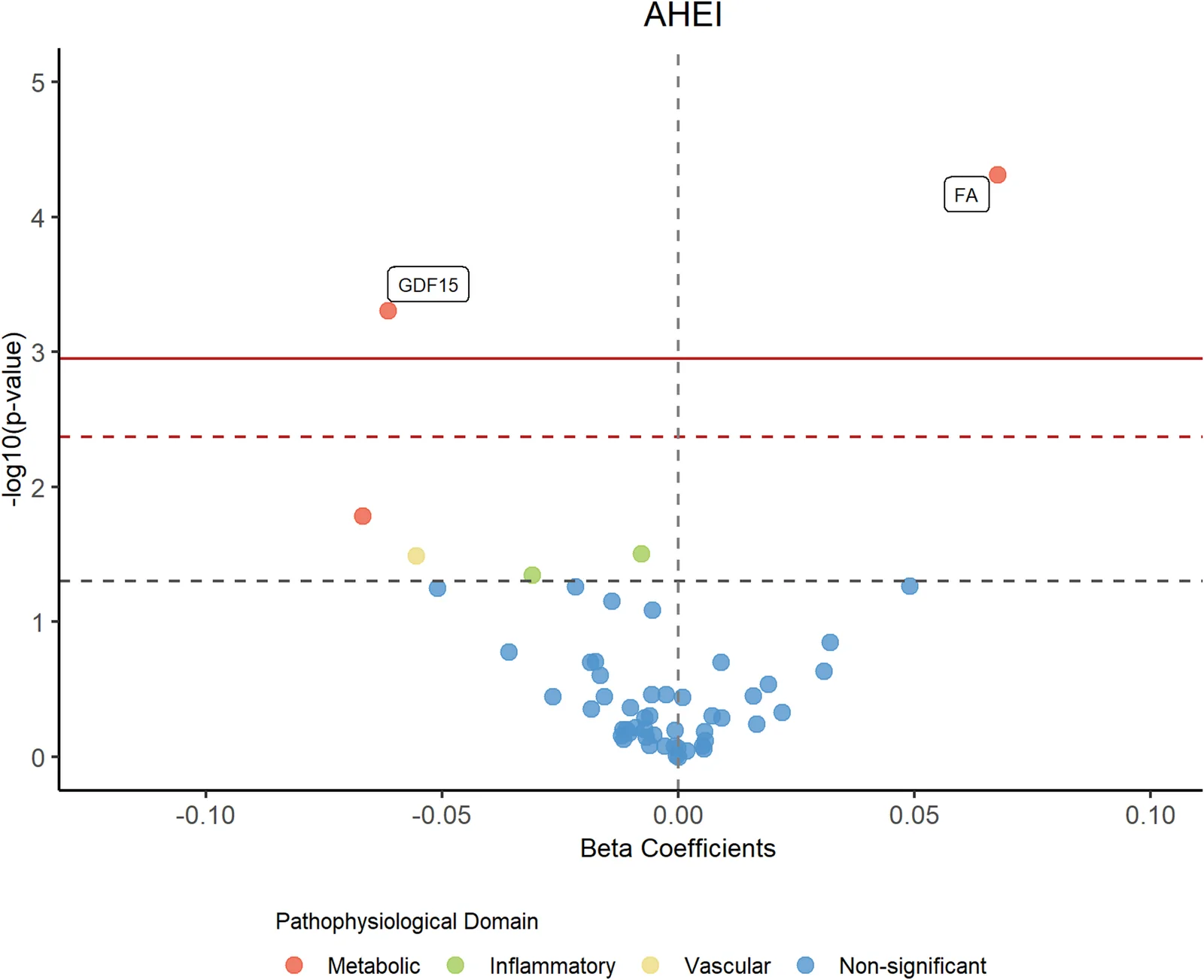
Volcano plots displaying the association between adherence to the Alternative Healthy Eating Index (AHEI) (per 1-SD increment) and blood-based biomarkers at the median (50th percentile) of the biomarkers’ distributions. Beta coefficients are taken from separate quantile regression models (one for each dietary pattern-biomarker combination) adjusted for age, sex, education, occupation, living arrangement, physical activity, smoking status, energy intake, and fasting status. The absolute value of each coefficient represents the strength of the association between adherence to a given dietary pattern and levels of a blood-based biomarker (across different pathophysiological domains), while the sign indicates the direction (direct or inverse) of the relationship. The horizontal dashed grey line indicates the threshold for statistical significance (p < 0.05). The horizontal dashed red line indicates the Benjamini-Hochberg corrected threshold for statistical significance (q < 0.10) and the solid red line indicates a more stringent threshold (q < 0.05). AHEI = Alternative Healthy Eating Index; FA = Folic acid; GDF 15 = Growth differentiation factor 15

**Fig. 3:**
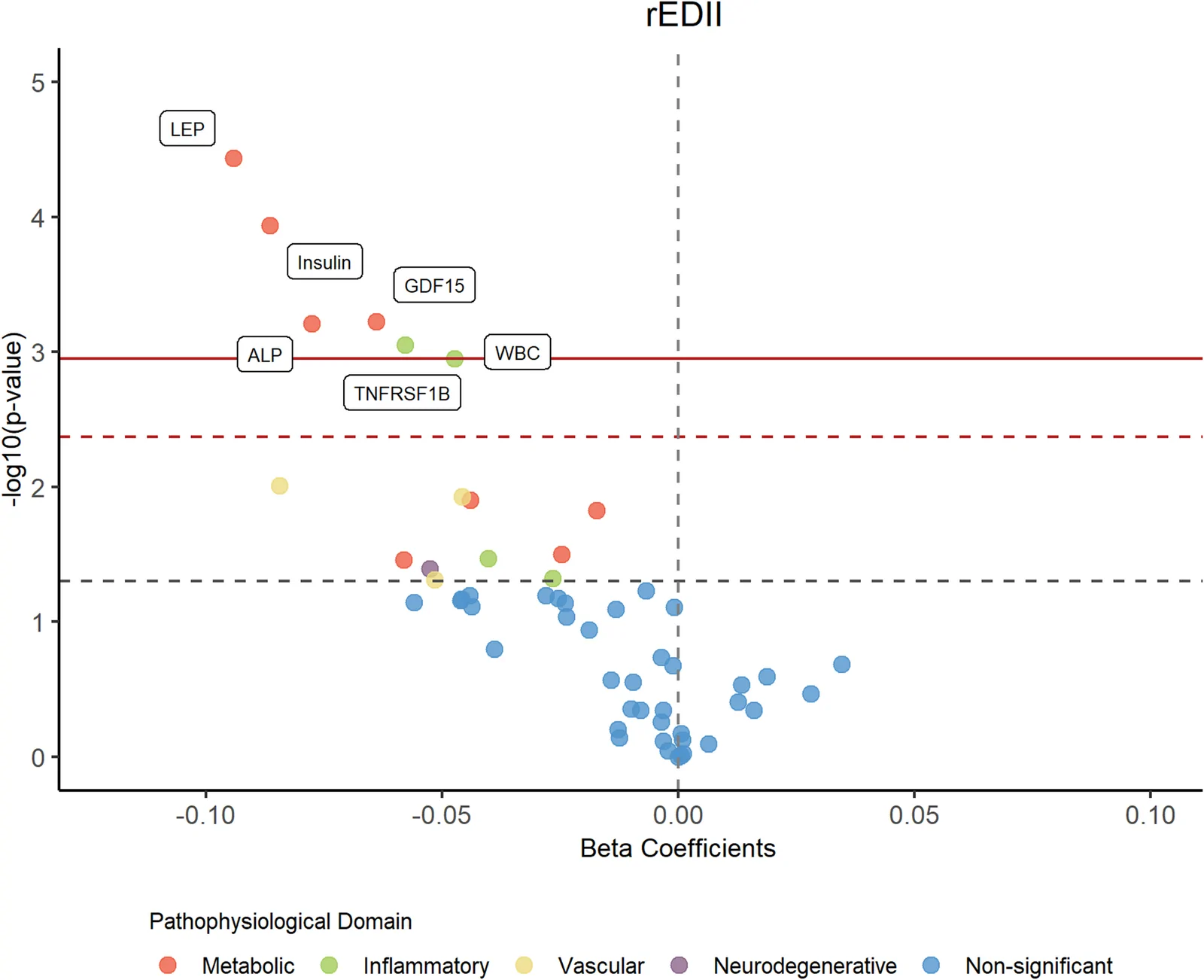
Volcano plots displaying the association between adherence to the reverse-transformed Empirical Dietary Inflammatory Index (rEDII) (per 1-SD increment) and blood-based biomarkers at the median (50th percentile) of the biomarkers’ distributions. Beta coefficients are taken from separate quantile regression models (one for each dietary pattern-biomarker combination) adjusted for age, sex, education, occupation, living arrangement, physical activity, smoking status, energy intake, and fasting status. The absolute value of each coefficient represents the strength of the association between adherence to a given dietary pattern and levels of a blood-based biomarker (across different pathophysiological domains), while the sign indicates the direction (direct or inverse) of the relationship. The horizontal dashed grey line indicates the threshold for statistical significance (p < 0.05). The horizontal dashed red line indicates the Benjamini-Hochberg corrected threshold for statistical significance (q < 0.10) and the solid red line indicates a more stringent threshold (q < 0.05). rEDII = reverse-transformed Empirical Dietary Inflammatory Index; ALP = Alkaline phosphatase; GDF 15 = Growth differentiation factor 15; LEP = Leptin; TNFRSF1B = Tumor necrosis factor receptor superfamily member 1B; WBC = Leukocytes

At the median biomarker levels, ten associations remained statistically significant after correction for multiple comparisons at the q < 0.1 threshold (Figs. 1, 2 and 3, Additional file 1: Table S7). Higher adherence to the AMED was associated with higher median folic acid [β (95% CI) = 0.066 (0.033, 0.099)] and lower median glial fibrillary acidic protein levels (GFAP) [-0.018 (-0.030, -0.006)]. Higher adherence to the AHEI was associated with higher median folic acid [0.068 (0.035, 0.100)] and lower median growth differentiation factor 15 (GDF15) levels [-0.061 (-0.096, -0.027)]. Higher adherence to the rEDII was associated with lower median levels of leptin [-0.094 (-0.139, -0.049)], insulin [-0.086 (-0.130, -0.043)], GDF15 [-0.064 (-0.100, -0.027)], alkaline phosphatase [-0.078 (-0.122, -0.033)], tumour necrosis factor receptor superfamily member 1B (TNFRSF1B) [-0.047 (-0.076, -0.019)], and leukocytes [-0.058 (-0.092, -0.024)].

### Sensitivity analyses

At the 25th percentile of biomarker levels, AMED adherence was associated with higher folic acid and lower gamma glutamyl transferase concentrations; AHEI adherence was linked to lower levels of gamma glutamyl transferase; and rEDII adherence was associated with lower levels of cystatin C, insulin, interleukin-6, interleukin-10, and chemokine CXC ligand 10(CXCL10). No significant associations were observed at the 75th percentile (Additional file 1: Fig. S3, S4, Table S7).

Most associations remained directionally consistent when the panel of standard biomarkers were re-examined at wave 3 (*n* = 1368), and even new significant associations emerged (Additional file 1: Fig. S5). Performing the analyses with participants who had complete case observations (*n* = 1619) rendered similar results to the main analyses (Additional file 1: Fig. S6). Restricting the analyses to participants with ≤ 4 chronic diseases (*n* = 1239) did not meaningfully impact the associations, except that of the rEDII with GDF15, which became nonsignificant (Additional file 1: Fig. S7). Additionally adjusting for number of medications yielded quite similar results (Additional file 1: Fig. S8), whereas separate additional adjustment for chronic disease burden rendered the association of the AHEI with GDF15 nominally significant, and those of the rEDII with TNFRSF1B and GDF15 nominally significant and nonsignificant, respectively (Additional file 1: Fig. S9). In addition to the significant associations identified in the main analysis, the inverse probability weighted analysis found that higher adherence to the AMED was associated with lower leukocyte and higher total—but not phosphorylated—tau levels; higher adherence to the AHEI was associated with lower β-2 microglobulin levels; and higher adherence to the rEDII was associated with lower cystatin C, CXCL10, gamma glutamyl transferase, C-peptide, vitamin B12, P-selectin, and C-C motif chemokine ligand 3 levels (Additional file 1: Fig. S10).

Sex-stratified analyses revealed only one significant diet×sex interaction after multiple comparison correction: AHEI adherence and CXCL10 (p-value for interaction = 0.001), driven by opposing directional associations in males (inverse) and females (direct) (Additional file 1: Fig. S11). Causal mediation analyses indicated that BMI yielded significant indirect effects (i.e., mediated) only for the association of rEDII adherence with insulin and leptin levels, whereas chronic disease burden showed significant indirect effects for rEDII adherence with levels of leukocytes, alkaline phosphatase, TNFRSF1B, insulin, leptin, and GDF15 (Additional file 1: Tables S8, S9).

## Discussion

In this cohort of older adults, we examined the associations between adherence to three healthy dietary patterns and a broad panel of blood-based biomarkers reflecting key metabolic, inflammatory, vascular, and neurodegenerative pathophysiological processes relevant to aging. Higher adherence to the AMED, AHEI, and rEDII was consistently associated with more favorable biomarker levels, after accounting for key socio-demographic and lifestyle confounders. Biomarkers showing the strongest associations with adherence to these dietary patterns—remaining statistically significant after multiple comparison correction—were higher folic acid (AMED, AHEI), lower GDF15 (AHEI, rEDII), lower GFAP (AMED), and lower leptin, insulin, alkaline phosphatase, TNFRSF1B, and leukocytes (rEDII). Associations remained consistent across multiple sensitivity analyses.

### Interpretation

Diet quality is increasingly being recognized as a determinant of healthy aging [37]. Previous studies have linked healthy dietary pattern adherence to metabolic (GDF15, adiponectin, folic acid, vitamin B12), inflammatory (C-reactive protein, interleukin-6, tumor necrosis factor α), vascular (intercellular adhesion molecule 1, vascular cell adhesion molecule 1, E-selectin) and neurodegenerative (phosphorylated tau 181, neurofilament light chain, GFAP) blood-based biomarkers [22, 30, 38–40, 19, 41]. However, most of these studies have typically focused on one dietary pattern at a time, assessed individual or fewer biomarkers at once, and only some were carried out specifically in older adult populations [22, 39, 41]. By leveraging dietary data from a well-characterized cohort of older adults and an extensive panel of blood-based biomarkers, this study advances current understanding of diet-biomarker relationships, extending them to several pathophysiological aging processes that have been only partially explored in previous research. We identified eight blood biomarkers showing the strongest associations with greater adherence to healthier dietary patterns. Most of the observed biomarkers associations were pattern-specific, suggesting that while all three dietary patterns are broadly considered healthy, they may influence aging through partially distinct biological processes owing to the synergistic effect of the specific foods each one emphasizes [4]. Notably, only two biomarkers remained significantly associated with more than one pattern: folic acid and GDF15.

Folic acid, a water-soluble B vitamin essential for one-carbon metabolism and DNA synthesis and repair, cannot be synthesized endogenously and must be obtained through diet or supplements, making it a reliable indicator of nutritional status [42]. Folic acid deficiency can lead to elevated homocysteine levels, a recognized risk factor for cognitive dysfunction and vascular diseases in older adults [43]. The strong, direct association between adherence to healthier, predominantly plant-based dietary patterns (AMED and AHEI) and higher folic acid levels is biologically plausible, given that the former emphasize folate-rich foods such as vegetables (including leafy green vegetables), legumes, and whole grains [44]. This is consistent with evidence that folic acid levels in older adults are highly sensitive to dietary intake given age-related reductions in absorption, making dietary quality—in the absence of mandatory fortification—a primary determinant in this population [43, 45]. GDF15, a stress-responsive cytokine upregulated in response to mitochondrial dysfunction, cellular stress, and inflammation, is an important biomarker associated with aging [46]. Elevated levels in older adults have been associated with a range of adverse age-related outcomes, including cardiometabolic diseases, decline in physical and cognitive function, and mortality [47]. The inverse association of adherence to the AHEI and rEDII with GDF15 levels is consistent with our previous findings in a Spanish cohort of older adults, and adds to the evidence that healthier diets and those with a lower inflammatory potential may attenuate underlying inflammation, possibly through the combined effects of antioxidants, polyphenols, and fibre-rich foods, which have previously been associated with downregulation of inflammatory pathways and lower risk of age-related diseases [19, 22]. However, the attenuation of the inverse rEDII-GDF15 association, both when restricting analyses to participants with fewer chronic diseases and following additional adjustment for chronic disease burden, alongside its partial mediation by the chronic disease count, suggests that reduced disease burden may be driving the observed association, rather than dietary inflammatory potential acting independently on lowering GDF15 levels [48]. The similar pattern observed following adjustment for number of medications is consistent with this interpretation, as medication use itself may be a downstream consequence of chronic disease burden [49]. Greater adherence to the AMED was associated with lower concentrations of GFAP, a marker of glial activation and neuroinflammation implicated in neurodegenerative diseases [50]. This finding corroborates our recent observations within the same cohort among participants aged ≥ 78 years, and aligns with emerging longitudinal evidence linking adherence to Mediterranean-style dietary patterns (higher consumption of nuts, olive oil, and fish) and lower levels of other neurodegenerative blood-biomarkers later in life [41, 51].

Adherence to the rEDII exhibited the broadest biological signature, with inverse associations across six biomarkers, pointing to possible links between dietary inflammatory potential and multiple interconnected biological processes, given the central role of inflammation in aging [52]. This may also in part reflect the empirical derivation of the EDII directly from inflammatory biomarker data, in contrast to the a priori food-component-based construction of the AMED and AHEI, which was not specifically calibrated to biomarker outcomes [29]. Regarding the observed rEDII associations, insulin and leptin are hormones involved in energy homeostasis, with insulin modulating glucose metabolism and leptin involved in appetite regulation [53]. In states of insulin and/or leptin resistance, disrupted metabolic signalling promotes the development of type 2 diabetes and obesity while impairing endothelial function and promoting atherosclerotic processes, collectively increasing the risk of cardiovascular diseases [54]. Alkaline phosphatase is an enzyme that serves as a useful clinical marker of bone disorders and hepatic dysfunction [55]. Elevated levels have also been linked to conditions such as chronic kidney disease, cardiovascular disease, and metabolic syndrome, reflecting pathological processes including vascular calcification, endothelial dysfunction, and systemic inflammation [56]. TNFRSF1B is a receptor that binds tumour necrosis factor α (a proinflammatory cytokine) and is upregulated in multiple prolonged and maladaptive inflammatory states [57]. Similarly, higher leukocyte counts are recognized as an indicator of chronic systemic inflammation, which accompanies immune system activation in several disease conditions [58]. The clustering of associations with metabolic and inflammatory biomarkers suggests that rEDII adherence relates to shared overlapping pathophysiological processes, which aligns with evidence that metabolic and immune related inflammatory pathologies are tightly coupled (e.g., through mechanisms such as insulin resistance and adipokine signalling), consistent with the hypothesis that dietary patterns with a lower inflammatory potential (low in trans and saturated fatty acids, added sugar and higher anti-inflammatory plant-based foods) may be associated with broader systemic process implicated in aging [59]. Furthermore, causal mediation analyses indicated that these associations were partly explained by adiposity and chronic disease burden, which is not surprising given their recognized roles as potential drivers of the pathophysiological states these biomarkers reflect [60].

Taken together, our findings support the concept that healthy dietary patterns are associated with multiple biological processes relevant to healthy aging, likely through different mechanisms that have yet to be fully elucidated. Such associations may parallel the observed benefits in epidemiological studies, where greater adherence to healthy dietary patterns has been linked to reduced risk of chronic disease, preserved cognitive and physical function, and improved mental well-being [37]. These benefits likely reflect both (1) the inclusion of nutrient-dense foods common across patterns (e.g., fruits, vegetables, whole grains, legumes, nuts, and fish) which provide fibre, antioxidants, polyphenols, essential micronutrients, and beneficial fatty acids, and (2) the concurrent reduction of less healthy components, including refined grains, sugar-sweetened beverages, and red and processed meats [15]. From a public health perspective, these results reinforce the importance of dietary guidelines that emphasize overall diet quality, achievable by combining beneficial elements of traditional diets (e.g., AMED), evidence-based disease-lowering dietary patterns (e.g., AHEI), and diets with lower inflammatory potential (e.g., rEDII), in order to enhance health outcomes while supporting cultural relevance and long-term adherence. This flexibility, alongside advances in precision nutrition, offers promising avenues for tailoring diets to individual aging-related health needs [15].

When analysing different percentiles of the biomarker distributions, the strongest dietary associations were near the median, with fewer associations at the extremes (particularly at the higher end). This suggests that diet is broadly associated with biological processes at both physiological and subclinical levels. Such patterns may be partly explained by the role of diet in modulating chronic low-grade inflammation, which often precedes overt clinical disease, pointing to the potential relevance of diet in delaying the onset of age-related diseases [61]. The single significant diet-sex interaction (AHEI adherence with CXCL10 levels) revealed opposing directional associations across sexes. One possible explanation might be the implication of higher CXCL10 (a proinflammatory chemokine) levels in autoimmune processes, which are disproportionately prevalent in women [62]. Such a predisposition may correspond to a sustained baseline CXCL10 elevation, reducing the range within which dietary quality is associated with potential beneficial effects in females relative to males. This is consistent with our quantile-specific finding that the rEDII-CXCL10 association was confined to the lower end of the biomarker distribution, suggesting that lower dietary inflammatory potential may be most relevant to CXCL10 at levels not yet driven by underlying pathophysiology. Other sex-specific associations in the absence of significant interactions should be interpreted cautiously, as they may partly reflect the unequal sample sizes across strata rather than a true sex-specific biological effect.

### Limitations

Although this study reports many findings, they should be interpreted in light of some limitations. The cross-sectional design precludes any causal inference and is susceptible to reverse causality, although the directional consistency of associations between diet quality and routinely measured biomarkers across waves 1 and 3 suggests it is less likely that biomarker status—or disease burden—was driving dietary intake. Residual confounding due to chronic diseases cannot be fully addressed through adjustment alone; although causal mediation analyses were conducted to partly address this, mediation estimates rely on untestable assumptions including the absence of unmeasured confounding and correct temporal ordering of exposure, mediator, and outcome. Dietary habits may also change with advancing age or socioeconomic circumstances, and although analyses were adjusted for these factors, residual confounding and temporal changes in diet cannot be fully excluded [63]. Despite using a validated FFQ, the most common dietary collection tool in large cohorts, recall errors and social desirability bias may influence reporting [25]. Additionally, equal dietary pattern adherence scores may mask heterogeneity in food and nutrient composition, potentially diluting specific diet–biomarker associations [6]. The absence of data on dietary supplement use limits our ability to determine the extent to which biomarker levels are attributable solely to dietary intake. Finally, the predominantly urban, highly educated, and affluent Swedish sample, with limited diversity in key social determinants, combined with the likelihood of missing data from older participants with higher disease burden, restricts the generalizability of these findings to other populations and settings. Although we conducted inverse probability weighting analyses to partially account for this potential source of bias, some residual selection bias may still remain.

## Conclusions

This study provides evidence that greater adherence to healthy dietary patterns is associated with more favourable metabolic and inflammatory blood-based biomarkers in older adults. Although the study design does not allow for causal inference, these findings reinforce the potential role of diet as a modifiable determinant of healthy aging. Future research employing longitudinal and interventional designs in larger, more diverse older populations is needed to confirm these findings, and it could inform the development of targeted clinical dietary strategies and precision public health interventions aimed at promoting healthy aging.

## Supplementary Information

Below is the link to the electronic supplementary material.

Supplementary Material 1

Supplementary Material 2

## Data Availability

Data are from the SNAC-K project, a population-based study designed to investigate aging processes and identify preventive strategies to enhance health and care in older adults. Access to these original data is available to the research community upon approval by the SNAC-K organization. Applications for accessing these data can be submitted through https://www.snac-k.se/. Analytical scripts for this study are available from the corresponding authors upon reasonable request.

## Abbreviations

AMED: Alternative Mediterranean Diet
AHEI: Alternative Healthy Eating Index
BMI: Body Mass Index
EDII: Empirical Dietary Inflammatory Index
FFQs: Food Frequency Questionnaires
GDF15: Growth Differentiation Factor 15
GFAP: Glial fibrillary acidic protein
rEDII: reverse-transformed Empirical Dietary Inflammatory Index
SD: Standard Deviation
SNAC-K: Swedish National Study on Aging and Care in Kungsholmen
TNFRSF1B: Tumour necrosis factor receptor superfamily member 1B
