# Supplementary material for: Biological signature of dietary patterns in older adults: findings from a population-based study
Source: BMC Med. 2026 Sep 15;24:520. doi: 10.1186/s12916-026-05202-2 (PMC13613825; doi:10.1186/s12916-026-05202-2)
Supplement: Supplementary file 1 [file 12916_2026_5202_MOESM1_ESM.docx]

**Additional file 1 (Supplementary material):** Biological signature of dietary patterns in older adults: findings from a cross-sectional study

**Table S1**: Median consumption of food components and scoring method of the Alternative Mediterranean Diet (AMED)

| Component | Total (N=1769) Median (IQR) |
| --- | --- |
| Vegetables (except potatoes) (g/day) | 164.5 (101.5 – 250.5) |
| Fruit (g/day) | 243.6 (134.9 – 366.8) |
| Nuts (g/day) | 0.05 (0.00 – 1.46) |
| Whole grains (g/day) | 51.2 (31.9 – 79.2) |
| Legumes (g/day) | 8.10 (4.05 – 12.9) |
| Fish (g/day) | 35.9 (25.5 – 49.8) |
| Monounsaturated to saturated fat (ratio) | 0.96 (0.84 – 1.08) |
| Red and processed meat (g/day) | 55.7 (35.4 – 80.8) |
| Alcohol (g of ethanol/day) | 8.90 (3.47 -18.5) |

To calculate the AMED score, a value of 0 or 1 was assigned to each of the nine groups, with the study population-specific median as the cutoff. For the components vegetables, fruits, nuts, whole grains, legumes, fish, and monounsaturated to saturated fat ratio, persons whose consumption was at or above the median were assigned a value of 1. For the component red and processed meat, persons whose consumption was below the median were assigned a value of 1. For alcohol intake, a value of 1 was assigned if consumed between 5 and 15 g of ethanol per day. The total score range was from 0 to 9.

**Table S2**: Mean consumption of food components and scoring criteria of the Alternative Healthy Eating Index (AHEI)

|  | | Criteria | | Mean (SD)  (N=1769) |
| --- | --- | --- | --- | --- |
| Component | | **For minimum score (0)** | **For maximum score (10)** |  |
| Vegetables (servings/day) | | 0 | ≥5 | 2.72 (2.05) |
| Fruit (servings/day) | | 0 | ≥4 | 2.09 (1.47) |
| Whole grains (g/day) | Males | 0 | 90 | 66.3 (42.3) |
|  | Females |  | 75 | 54.6 (33.6) |
| Nuts and legumes (servings/day) | | 0 | ≥1 | 0.10 (0.88) |
| Long chain omega-3 fatty acids (DHA, EPA) (mg/day) | | 0 | 250 | 356.1 (262.9) |
| PUFA (% of energy/day) | | ≤2 | ≥10 | 5.31 (1.46) |
| Alcohol (drinks/day) | Males | ≥2.5 | 0.5-2.0 | 1.83 (1.56) |
|  | Females |  | 0.5-1.5 | 0.91 (0.99) |
| Sugar-sweetened beverages and fruit juice (servings/day) | | ≥1 | 0 | 0.27 (0.54) |
| Red and processed meat (servings/day) | | ≥1.5 | 0 | 0.95 (0.79) |
| Trans fat (% of energy/day) | | ≥4 | ≤0.5 | 1.27 (0.43) |
| Sodium (mg/day) | | Highest decile | Lowest decile | 2539.7 (1056.9) |

**Vegetables**: one serving is 0.5 cup of vegetables/1 cup of green leafy vegetables (1 cup = 236.59 g); **Fruit**: one serving is 1 medium piece of fruit or 0.5 cup of berries (1 cup = 236.59 g); **Whole grains**: one serving of a 100% whole-grain product (i.e., 0.5 cup of oatmeal or brown rice) contains15–20 g of whole grains (per dry weight); **Nuts, legumes, and vegetable protein**: one serving is 1 oz (1 oz = 28.35 g) of nuts or 1 tablespoon (15 mL) of peanut butter; **Polyunsaturated fatty acids (PUFA)**: the highest score to individuals with 10% of total energy intake from PUFA. PUFA does not include eicosapentaenoic acid (EPA) or docosahexaenoic acid (DHA) intake; **Alcohol**: non-drinkers received a score of 2.5. Because the health effects of alcohol are seen at lower quantities in females than in males, we used sex-specific cutoffs. One drink is 4 oz of wine, 12 oz of beer, or 1.5 oz of liquor (1 oz = 28.35 g); **Sugar-sweetened beverages**: one serving is 8 oz (1 oz = 28.35 g); **Red meat and processed meat**: one serving is 4 oz of unprocessed meat or 1.5 oz of processed meat (1 oz = 28.35 g); **Trans-isomers**: Cutoffs are consistent with original AHEI cutoffs for trans-fat; **Sodium**: Values in lowest decile were below 1421 mg/d and in highest decile were above 3796 mg/d.

**Table S3**: Mean consumption of food components, overall inflammatory effect scores, and scoring method of the Empirical Dietary Inflammatory Index (EDII)

| Components | Mean (SD)  N=1769 | Overall inflammatory effect score |
| --- | --- | --- |
| Pro-inflammatory EDII components (portions/day) ^1^ |  |  |
| Processed meat | 0.64 (0.69) | 165.03 |
| Red meat | 0.27 (0.21) | 140.19 |
| Organ meat | 0.03 (0.04) | 144.61 |
| Fish (other than dark-meat fish) | 0.20 (0.15) | 252.45 |
| Vegetables (other than dark yellow and leafy green) | 0.80 (0.65) | 136.14 |
| Refined grains | 1.30 (0.97) | 81.21 |
| High-energy beverages | 0.27 (0.54) | 156.85 |
| Low-energy beverages | 0.03 (0.09) | 94.77 |
| Tomatoes | 0.64 (0.62) | 167.92 |
| Anti-inflammatory EDII components (portions/day) ^2^ |  |  |
| Beer | 0.34 (0.51) | –136.99 |
| Wine | 0.32 (0.37) | –249.70 |
| Tea | 0.66 (0.82) | –42.25 |
| Coffee | 1.69 (1.08) | –83.18 |
| Dark yellow vegetables | 0.41 (0.48) | –165.37 |
| Leafy green vegetables | 0.58 (0.59) | –190.29 |
| Snacks | 0.14 (0.21) | –45.08 |
| Fruit juice | 0.37 (0.502) | –58.95 |
| Pizza | 0.02 (0.04) | –1175.21 |

Each participant’s mean daily consumption of each food component was multiplied by its corresponding inflammatory effect score, summed across all components to derive the EDII score, and standardised by dividing by 100.

^1^**Processed meat**: processed meats, bacon, or hot dog; **Red meat**: beef, pork, lamb, or hamburger patty; **Organ meat**: beef, calf, or pork liver, or chicken or turkey liver; **Fish** (other than dark-meat fish): canned tuna, shrimp, lobster, scallops, fish, or other seafood other than dark-meat fish; **Other vegetables**: celery, mushrooms, green pepper, corn, mixed vegetables, eggplant, zucchini, alfalfa sprouts, or cucumber; **Refined grains**: white bread; English muffin, bagel or roll, muffin or biscuit, pancakes, or waffles; or white rice; or pasta; **High-energy beverages**: cola with sugar, other carbonated beverages with sugar, or fruit punch drinks; **Low-energy beverages**: low-energy cola or other low-energy carbonated beverages; **Tomatoes**: fresh tomato, tomato juice, or tomato sauce.

^2^ **Wine**: red or white wine; **Coffee**: soluble, espresso, Italian, or filtered coffee; **Dark yellow vegetables**: carrots, yellow (winter) squash, yams, or sweet potatoes; **Leafy green vegetables**: cooked or raw spinach, iceberg or head lettuce, or romaine or leaf lettuce; **Snacks**: potato chips, corn chips, popcorn, or crackers; **Fruit juice**: apple juice or cider, orange juice, grapefruit juice, or other fruit juice.

**Table S4**: Biomarker names, acronyms and analytical platform

|  | Acronyms | Biomarkers | Concentrate (pg/mL) | Intra /Inter CV (%) | Reagents | Analytical platform |
| --- | --- | --- | --- | --- | --- | --- |
| 1 | Aβ40 | Amyloid beta protein 40 | 117.5 | 2.2/5.0 | Neuro 3-plex A;101995;Lot 503659 | Quanterix Simoa SR-X |
| 2 | Aβ42 | Amyloid beta protein 42 | 6.6 | 4.8/7.1 | Neuro 3-plex A;101995;Lot 503659 | Quanterix Simoa SR-X |
| 3 | Adipo | Adiponectin | 18.6 *10^6^ | 9.4/13.2 | RnD Systems; LXSAHM-02;Lot L146268 | Luminex. FlexMap 3D |
| 4 | ALB | Albumin | N/A | N/A | N/A | N/A |
| 5 | ALP | Alkaline phosphatase | N/A | N/A | N/A | N/A |
| 6 | aSyn | Alpha synuclein | 1884 | 3.7/6.5 | RnD Systems; LXSAHM-18; Lot L146266 | Luminex. FlexMap 3D |
| 7 | BDNF | Brain-derived neurotrophic factor | 26.3 *10^3^ | 15.1/11.1 | RnD Systems; LXSAHM-04; Lot L146267 |  |
| 8 | B2M | b-2-microglobulin | 2.8*10^6^ | 7.8/13.7 | RnD Systems;LXSAHM-01;Lot L146270 | Luminex. FlexMap 3D |
| 9 | Ca | Calcium | N/A | N/A | N/A | N/A |
| 10 | CCL11 | C-C motif chemokine 11 | 369 | 4.7/7.6 | RnD Systems; LXSAHM-18; Lot L146266 | Luminex FlexMap 3D |
| 11 | CCL2 | C-C motif chemokine 2 | 12.4 *10^3^ | 10.6/10.1 | RnD Systems; LXSAHM-02; L146269 | Luminex FlexMap 3D |
| 12 | CCL3 | C-C motif chemokine 3 | 11.2*10^3^ | 3.8/8.4 | RnD Systems; LXSAHM-18; Lot L146266 | Luminex FlexMap 3D |
| 13 | CCL4 | C-C motif chemokine 4 | 2.0 *10^3^ | 6.7/11.2 | RnD Systems; LXSAHM-18; Lot L146266 | Luminex FlexMap 3D |
| 14 | CPEP | C-peptide | 2.3 *10^3^ | 3.9/12.3 | RnD Systems; LXSAHM-18; Lot L146266 | Luminex. FlexMap 3D |
| 15 | Cr | Creatinine | N/A | N/A | N/A | N/A |
| 16 | CRP | C-reactive protein | 18.6 *10^6^ | 14.2/11 | RnD Systems; LXSAHM-02;Lot L146268 | Luminex FlexMap 3D |
| 17 | CXCL10 | Chemokine CXC ligand 10 | 61.0 | 8.9/6.2 | RnD Systems; LXSAHM-18; Lot L146266 | Luminex FlexMap 3D |
| 18 | CysC | Cystatin C | 890.9 *10^3^ | 6.1/5.9 | RnD Systems; LXSAHM-04; Lot L146267 | Luminex FlexMap 3D |
| 19 | EGF | Epidermal growth factor | 219.0 | 12.8/14.2 | RnD Systems; LXSAHM-18; Lot L146266 | Luminex FlexMap 3D |
| 20 | EphA2 | Ephrin type-A receptor 2 | 2.6 *10^3^ | 5.7/9.2 | RnD Systems; LXSAHM-18; Lot L146266 | Luminex FlexMap 3D |
| 21 | ESel | E-Selectin | 32.4 *10^3^ | 15.7/7.0 | RnD Systems; LXSAHM-18; Lot L146266 | Luminex FlexMap 3D |
| 22 | FA | Folic acid | N/A | N/A | N/A | N/A |
| 23 | GDF15 | Growth differentiation factor 15 | 1157 | 16.0/12.3 | RnD Systems; LXSAHM-18; Lot L146266 | Luminex. FlexMap 3D |
| 24 | GFAP | Glial fibrillary acidic protein | 194.0 | 13.1/21.4 | Neuro 2-plex B; 103520; Lot 503409 | Quanterix Simoa SR-X |
| 25 | GGT | Gamma-glutamyl transferase | N/A | N/A | N/A | N/A |
| 26 | Hb | Hemoglobin | N/A | N/A | N/A | N/A |
| 27 | HbA1c | Hemoglobin A1c | N/A | N/A | N/A | N/A |
| 28 | ICAM1 | Intercellular Adhesion Molecule 1 | 648 *103 | 4.3/9.3 | RnD Systems; LXSAHM-18; Lot L146266 | Luminex FlexMap 3D |
| 29 | IFNγ | Interferon gamma | N/A | N/A | CorPlex Human Cytokine Panel 1 | Quanterix Simoa SP-X |
| 30 | IGFBP1 | Insulin-like growth factor binding protein 1 | 27.1 *10^3^ | 4.7/4.7 | RnD Systems; LXSAHM-18; Lot L146266 | Luminex FlexMap 3D |
| 31 | IL-1 β | Interleukin 1 beta | N/A | N/A | CorPlex Human Cytokine Panel 1 | Quanterix Simoa SP-X |
| 32 | IL-10 | Interleukin 10 | N/A | N/A | CorPlex Human Cytokine Panel 1 | Quanterix Simoa SP-X |
| 33 | IL-12p70 | Interleukin 12p70 | N/A | N/A | CorPlex Human Cytokine Panel 1 | Quanterix Simoa SP-X |
| 34 | IL-6 | Interleukin 6 | N/A | N/A | CorPlex Human Cytokine Panel 1 | Quanterix Simoa SP-X |
| 35 | IL-8 | Interleukin 8 | N/A | N/A | CorPlex Human Cytokine Panel 1 | Quanterix Simoa SP-X |
| 36 | Insulin | Insulin | 1210 | 4.2/12.2 | RnD Systems; LXSAHM-18; Lot L146266 | Luminex. FlexMap 3D |
| 37 | LEP | Leptin | 20.9*10^3^ | 11.3/12.3 | RnD Systems; LXSAHM-18; Lot L146266 | Luminex. FlexMap 3D |
| 38 | MPO | Myeloperoxidase | 144.1 *10^3^ | 15.6//12.8 | RnD Systems; LXSAHM-04; Lot L146267 | Luminex FlexMap 3D |
| 39 | NCAD | N-Cadherin | 513.6*10^3^ | 15.3/ 5.1 | RnD Systems; LXSAHM-04; Lot L146267 | Luminex FlexMap 3D |
| 40 | NfL | Neurofilament light chain | 24.1 | 7.6/11.6 | Neuro 2-plex B; 103520; Lot 503409 | Quanterix Simoa SR-X |
| 41 | PSel | P-selectin | 54.1*10^3^ | 7.1/28.8 | RnD Systems; LXSAHM-18; Lot L146266 | Luminex FlexMap 3D |
| 42 | ptau181 | phosphorylated tau 181 | 1.4 | 9.2/14.4 | Simoa pTau181 Advantage V2 Kit | Quanterix Simoa SR-X |
| 43 | ptau217 | phosphorylated tau 217 | 0.14 | 11/13.4 | Simoa ALZpath p-Tau217 Advantage PLUS | Quanterix Simoa SR-X |
| 44 | S100B | S100 calcium-binding protein B | 1334 | 6.9/25.5 | RnD Systems; LXSAHM-18; Lot L146266 | Luminex FlexMap 3D |
| 45 | T4 | Thyroxine | N/A | N/A | N/A | N/A |
| 46 | TC | Total cholesterol | N/A | N/A | N/A | N/A |
| 47 | TNFα | Tumor necrosis factor alpha | N/A | N/A | N/A | N/A |
| 48 | TNFRSF1B | Tumor necrosis factor receptor superfamily member 1B | 5340 | 14.5/23 | RnD Systems; LXSAHM-02; L146269 | Luminex FlexMap 3D |
| 49 | TSH | Thyroid stimulating hormone | N/A | N/A | N/A | N/A |
| 50 | ttau | Total tau | 0.9 | 10.7/11.9 | Neuro 3-plex A;101995;Lot 503659 | Quanterix Simoa SR-X |
| 51 | VCAM1 | Vascular cell adhesion protein 1 | 834 *10^3^ | 18.0/17.0 | RnD Systems; LXSAHM-18; Lot L146266 | Luminex FlexMap 3D |
| 52 | VEGF | Vascular endothelial growth factor | 172 | 8.3/5.9 | RnD Systems; LXSAHM-18; Lot L146266 | Luminex FlexMap 3D |
| 53 | VitB12 | Vitamin B 12 | N/A | N/A | N/A | N/A |
| 54 | WBC | Leucocytes | N/A | N/A | N/A | N/A |

CV: coefficient of variation. N/A: Information on the specific analytical platforms used to measure routine laboratory biomarkers is not available; however, measurement was conducted in accordance with standardized institutional protocols, at the hematological and biochemical laboratory at Karolinska Hospital.

**Table S5:** Sources of missing biomarker data among participants with available blood sample (N=2529).

| Platform | Participants missing ≥1 biomarker (N) | Participants with missing value for each biomarker (N) | Missingness reason | Data handling notes* |
| --- | --- | --- | --- | --- |
| Routine laboratory biomarkers | 161/2529 | T4, TSH (129 each); VitB12 (17); HbA1c (16); TC, FA (15 each); ALP, GGT (14 each); WBC, Ca, Cr (13 each); ALB (11); Hb (10) | Not recorded | - |
| AD biomarkers | 169/2529 | ptau217 (169); ptau181 (85); Aβ40, Aβ42, ttau (14 each); NfL, GFAP (12 each) | Insufficient /no volume left; ptau217 added later | Values below LOD for AD biomarkers were imputed to lowest measured value (0): ttau (n=16), ptau181 (n=15), Aβ42 (n=7), ptau217 (n=5), Aβ40 (n=1).  1 participant with extreme ptau181 value (239.7 pg/mL; ~38× higher than 99th percentile) was considered an outlier and counted as missing. |
| Luminex panel biomarkers | 38/2529 | Adipo, BDNF, B2M, CRP, CysC, MPO, NCAD (36 each); CCL2, TNFRSF1B, VCAM (37 each); aSyn, CCL3, CCL4, CCL11, CPEP, CXCL10, EGF, EphA2, ESel, GDF15, ICAM1, IGFBP1, Insulin, LEP, PSel, S100B, VEGF (38 each) | Insufficient volume/ no volume left | For participants with ≤6 missing Luminex panel biomarkers, values were imputed to LOD: B2M (n=35), BDNF, CCL2, TNFRSF1B, CysC (n=4 each), Adipo (n=20), CRP (n=17), NCAD, MPO (n=6 each), EGF (n=69), VEGF (n=7) |
| Cytokine panel biomarkers | 20/2529 | IFNγ, IL-1β, IL-6, IL-8, IL-10, IL-12p70, TNFα (20 each) | Insufficient volume/ no volume left | For participants with ≤1 missing cytokine panel biomarkers, values were imputed to the lowest measured value as LOD was unavailable: IFNγ, IL-1β (n=3 each), IL-6 (n=2), IL-12p70 (n=140) |

* Imputed values were retained in the analysis and were not counted as missing.
Columns 2 and 3 shows the number of participants with ≥1 missing biomarker within each platform. Counts are not mutually exclusive because participants may have missing biomarkers in multiple platforms. Among the 328 participants with ≥1 missing biomarker, the mutually exclusive distribution was: routine laboratory only (N=154); AD only (N=119); Luminex only (N=4); Cytokines only (N=1); routine laboratory + AD (N=5); AD + Luminex (N=25); AD + Cytokines (N=10); routine + AD + Cytokines (N=1); routine + AD + Luminex (N=1); AD + Luminex + Cytokines (N=8)

N = number of participants; n = number of imputed measurements; LOD = Lowest limit of detection; AD = Alzheimer’s disease
Aβ40 = Amyloid beta protein 40; Aβ42 = Amyloid beta protein 42; Adipo = Adiponectin; ALB = Albumin; ALP = Alkaline phosphatase; aSyn = Alpha synuclein; B2M = β-2-microglobulin; BDNF = Brain-derived neurotrophic factor; Ca = Calcium; CCL2 = C-C motif chemokine ligand 2; CCL3 = C-C motif chemokine ligand 3; CCL4 = C-C motif chemokine ligand 4; CCL11 = C-C motif chemokine ligand 11; CPEP = C-peptide; Cr = Creatinine; CRP = C-reactive protein; CXCL10 = C-X-C motif chemokine ligand 10; CysC = Cystatin C; EGF = Epidermal growth factor; EphA2 = Ephrin type-A receptor 2; ESel = E-selectin; FA = Folic acid; GDF15 = Growth differentiation factor 15; GFAP = Glial fibrillary acidic protein; GGT = Gamma-glutamyl transferase; Hb = Hemoglobin; HbA1c = Hemoglobin A1c; ICAM1 = Intercellular adhesion molecule 1; IFNγ = Interferon gamma; IGFBP1 = Insulin-like growth factor-binding protein 1; IL-1β = Interleukin 1 beta; IL-6 = Interleukin 6; IL-8 = Interleukin 8; IL-10 = Interleukin 10; IL-12p70 = Interleukin 12p70; LEP = Leptin; MPO = Myeloperoxidase; NCAD = N-cadherin; NfL = Neurofilament light chain; PSel = P-selectin; ptau181 = Phosphorylated tau 181; ptau217 = Phosphorylated tau 217; S100B = S100 calcium-binding protein B; T4 = Thyroxine; TC = Total cholesterol; TNFα = Tumor necrosis factor alpha; TNFRSF1B = Tumor necrosis factor receptor superfamily member 1B; TSH = Thyroid-stimulating hormone; ttau = Total tau; VCAM1 = Vascular cell adhesion protein 1; VEGF = Vascular endothelial growth factor; VitB12 = Vitamin B12; WBC = Leukocytes

**Table S6:** Characteristics of participants in the analytical sample and excluded samples.

|  | Analytical sample  (N=1769) N (%) | Excluded sample  (N=1594) N (%) |
| --- | --- | --- |
| Age * |  |  |
| 60 to <65 years | 560 (31.7) | 179 (11.2) |
| 65 to <70 years | 360 (20.3) | 205 (12.9) |
| 70 to <80 years | 525 (29.7) | 414 (26.0) |
| ≥ 80 years | 324 (18.3) | 796 (49.9) |
| Sex * |  |  |
| Male | 707 (40.0) | 474 (29.7) |
| Female | 1062 (60.0) | 1120 (70.3) |
| Living arrangement |  |  |
| Alone | 873 (49.3) | 377 (23.6) |
| Not alone | 896 (50.7) | 327 (20.5) |
| *Missing* | 0 | 890 |
| Previous occupation * |  |  |
| Manual worker | 343 (19.4) | 447 (28.0) |
| Non-manual worker | 1426 (80.6) | 1066 (66.9) |
| *Missing* | 0 | 81 |
| Education * |  |  |
| Elementary | 229 (12.9) | 361 (22.6) |
| High school | 864 (48.8) | 787 (49.4) |
| University | 676 (38.3) | 414 (25.9) |
| *Missing* | 0 | 32 |
| Tobacco smoking * |  |  |
| Never | 752 (42.5) | 643 (40.3) |
| Former smoker | 714 (40.4) | 460 (28.9) |
| Current smoker | 278 (15.7) | 169 (10.6) |
| *Missing* | 25 | 322 |
| Physical activity |  |  |
| Inadequate | 305 (17.2) | 131 (8.22) |
| Health-enhancing | 891 (50.4) | 362 (22.7) |
| Fitness-enhancing | 465 (26.3) | 154 (9.66) |
| *Missing* | 108 | 947 |
| Energy intake |  |  |
| <1500 kcal/day | 442 (25.0) | 180 (11.3) |
| 1500-2000 kcal/day | 542 (30.6) | 240 (15.1) |
| ≥ 2000 kcal/day | 785 (44.4) | 284 (17.8) |
| *Missing* | 0 | 890 |
| Body mass index (BMI) * |  |  |
| <18.5 kg/m^2^ | 27 (1.50) | 62 (3.90) |
| 18.5 – 24.9 kg/m^2^ | 761 (43.0) | 603 (37.8) |
| 25 – 29.9 kg/m^2^ | 727 (41.1) | 474 (29.7) |
| ≥30 kg/m^2^ | 239 (13.5) | 150 (9.40) |
| *Missing* | 15 | 305 |
| No. of chronic diseases * |  |  |
| 0 – 1 | 292 (16.5) | 120 (7.53) |
| 2 – 3 | 681 (38.5) | 446 (27.9) |
| 4 – 5 | 476 (26.9) | 465 (29.2) |
| ≥ 6 | 320 (18.1) | 563 (35.3) |
| No. of medications * |  |  |
| 0 – 1 | 576 (32.6) | 317 (19.9) |
| 2 – 4 | 670 (37.8) | 500 (31.4) |
| ≥ 5 | 523 (29.6) | 762 (47.8) |
| *Missing* | 0 | 15 |

* Indicates a statistically significant difference between the samples according to chi-square tests (p < 0.05).

**Table S7:** Association between dietary pattern adherence (per 1-SD increment) and blood-based biomarkers (at the 25th, 50th, and 75th percentiles of the biomarkers’ distribution).

|  |  | AMED [β (95% CI)] |  |  | AHEI [β (95% CI)] |  |  | rEDII [β (95% CI)] |  |
| --- | --- | --- | --- | --- | --- | --- | --- | --- | --- |
|  | 25th percentile | 50th percentile | 75th percentile | 25th percentile | 50th percentile | 75th percentile | 25th percentile | 50th percentile | 75th percentile |
| Metabolism |  |  |  |  |  |  |  |  |  |
| Adiponectin | 0.025 (-0.020, 0.071) | 0.042 (-0.009, 0.093) | 0.012 (-0.060, 0.084) | 0.023 (-0.022, 0.068) | 0.049 (-0.001, 0.099) | 0.018 (-0.051, 0.086) | 0.013 (-0.033, 0.059) | 0.006 (-0.043, 0.056) | -0.019 (-0.087, 0.048) |
| Albumin | -0.038 (-0.109, 0.032) | -0.045 (-0.107, 0.017) | 0.019 (-0.045, 0.083) | 0.006 (-0.062, 0.075) | -0.012 (-0.073, 0.049) | 0.016 (-0.046, 0.078) | 0.016 (-0.053, 0.086) | 0.001 (-0.060, 0.061) | -0.017 (-0.079, 0.045) |
| Alkaline phosphatase | -0.017 (-0.062, 0.028) | -0.022 (-0.070, 0.027) | -0.038 (-0.105, 0.030) | -0.017 (-0.063, 0.028) | -0.012 (-0.059, 0.036) | -0.042 (-0.109, 0.025) | -0.027 (-0.073, 0.018) | -0.078 (-0.122, -0.033)*** | -0.092 (-0.157, -0.026)* |
| Calcium | 0.002 (-0.060, 0.064) | 0.012 (-0.042, 0.065) | -0.045 (-0.108, 0.018) | 0.024 (-0.035, 0.083) | -0.006 (-0.058, 0.046) | -0.007 (-0.068, 0.055) | -0.009 (-0.068, 0.050) | -0.013 (-0.064, 0.039) | 0.010 (-0.051, 0.071) |
| C-peptide | 0.009 (-0.037, 0.055) | -0.030 (-0.086, 0.026) | -0.003 (-0.080, 0.074) | -0.016 (-0.061, 0.029) | -0.067 (-0.121, -0.012)* | -0.036 (-0.115, 0.043) | -0.018 (-0.064, 0.027) | -0.058 (-0.112, -0.004)* | -0.063 (-0.138, 0.012) |
| Creatinine | 0.021 (-0.008, 0.050) | 0.005 (-0.024, 0.033) | 0.007 (-0.036, 0.050) | 0.017 (-0.012, 0.045) | -0.018 (-0.044, 0.009) | -0.019 (-0.062, 0.024) | -0.008 (-0.038, 0.022) | -0.024 (-0.051, 0.004) | -0.039 (-0.080, 0.001) |
| Cystatin C | -0.007 (-0.036, 0.023) | -0.004 (-0.039, 0.031) | -0.004 (-0.052, 0.043) | -0.021 (-0.048, 0.006) | -0.009 (-0.043, 0.025) | -0.039 (-0.086, 0.008) | -0.044 (-0.072, -0.017)** | -0.044 (-0.079, -0.009)* | -0.053 (-0.101, -0.005)* |
| Folic acid | 0.042 (0.019, 0.065)*** | 0.066 (0.033, 0.099)*** | 0.052 (-0.019, 0.122) | 0.031 (0.009, 0.054)* | 0.068 (0.035, 0.100)*** | 0.022 (-0.050, 0.094) | 0.020 (-0.001, 0.040) | 0.019 (-0.014, 0.051) | -0.040 (-0.114, 0.033) |
| γGT | -0.020 (-0.033, -0.007)** | -0.016 (-0.039, 0.007) | -0.040 (-0.086, 0.006) | -0.019 (-0.032, -0.007)** | -0.022 (-0.044, 0.0004) | -0.033 (-0.079, 0.013) | -0.010 (-0.023, 0.002) | -0.025 (-0.047, -0.002)* | -0.054 (-0.100, -0.008)* |
| GDF 15 | -0.002 (-0.036, 0.033) | -0.013 (-0.050, 0.024) | -0.030 (-0.084, 0.023) | -0.033 (-0.065, -0.001)* | -0.061 (-0.096, -0.027)*** | -0.064 (-0.115, -0.013)* | -0.035 (-0.067, -0.003)* | -0.064 (-0.100, -0.027)*** | -0.086 (-0.139, -0.033)* |
| Hemoglobin | -0.005 (-0.063, 0.054) | 0.002 (-0.047, 0.051) | 0.017 (-0.031, 0.065) | 0.018 (-0.038, 0.074) | -0.010 (-0.059, 0.038) | 0.010 (-0.037, 0.057) | -0.030 (-0.086, 0.025) | -0.044 (-0.092, 0.005) | -0.021 (-0.069, 0.026) |
| Insulin | 0.019 (-0.009, 0.047) | 0.017 (-0.030, 0.064) | -0.028 (-0.111, 0.056) | -0.014 (-0.041, 0.014) | -0.011 (-0.056, 0.034) | -0.054 (-0.138, 0.031) | -0.040 (-0.066, -0.013)** | -0.086 (-0.130, -0.043)*** | -0.109 (-0.189, -0.028)* |
| HbA1c | 0.013 (-0.026, 0.051) | 0.018 (-0.016, 0.052) | 0.005 (-0.040, 0.051) | -0.007 (-0.046, 0.032) | 0.016 (-0.018, 0.049) | -0.014 (-0.057, 0.030) | -0.018 (-0.056, 0.021) | 0.001 (-0.033, 0.035) | -0.044 (-0.089, 0.0002) |
| IGFBP1 | -0.003 (-0.017, 0.011) | -0.008 (-0.035, 0.018) | -0.019 (-0.072, 0.035) | -0.008 (-0.023, 0.007) | -0.010 (-0.035, 0.015) | -0.009 (-0.061, 0.044) | 0.006 (-0.009, 0.020) | 0.013 (-0.012, 0.038) | 0.012 (-0.040, 0.064) |
| Leptin | 0.004 (-0.033, 0.042) | 0.005 (-0.043, 0.052) | 0.011 (-0.065, 0.087) | -0.019 (-0.054, 0.017) | -0.018 (-0.065, 0.028) | -0.007 (-0.080, 0.067) | -0.047 (-0.082, -0.012)* | -0.094 (-0.139, -0.049)*** | -0.097 (-0.169, -0.025)* |
| T4 | 0.000 (-0.036, 0.036) | 0.000 (-0.051, 0.051) | 0.030 (-0.047, 0.106) | 0.000 (-0.035, 0.035) | 0.000 (-0.049, 0.049) | 0.013 (-0.062, 0.087) | 0.000 (-0.035, 0.035) | 0.000 (-0.049, 0.049) | -0.026 (-0.100, 0.048) |
| TSH | 0.031 (-0.003, 0.065) | 0.037 (0.001, 0.073)* | 0.015 (-0.032, 0.063) | 0.017 (-0.014, 0.048) | 0.019 (-0.016, 0.054) | 0.029 (-0.018, 0.076) | 0.010 (-0.021, 0.042) | -0.002 (-0.036, 0.032) | -0.010 (-0.057, 0.036) |
| Total cholesterol | -0.069 (-0.129, -0.008)* | -0.036 (-0.093, 0.021) | -0.042 (-0.108, 0.025) | -0.051 (-0.114, 0.012) | -0.027 (-0.083, 0.030) | -0.042 (-0.105, 0.022) | 0.012 (-0.053, 0.077) | 0.035 (-0.019, 0.088) | 0.043 (-0.020, 0.107) |
| Vitamin B12 | 0.014 (0.003, 0.025)* | 0.016 (0.003, 0.030)* | 0.008 (-0.018, 0.034) | 0.005 (-0.006, 0.017) | 0.009 (-0.005, 0.023) | 0.010 (-0.015, 0.036) | -0.014 (-0.024, -0.003)* | -0.017 (-0.031, -0.003)* | -0.036 (-0.062, -0.009)* |
| Inflammation |  |  |  |  |  |  |  |  |  |
| β2- microglobulin | 0.002 (-0.019, 0.024) | -0.010 (-0.040, 0.021) | -0.040 (-0.083, 0.004) | -0.004 (-0.026, 0.018) | -0.031 (-0.061, -0.001)* | -0.052 (-0.095, -0.009)* | -0.011 (-0.032, 0.010) | -0.028 (-0.058, 0.002) | -0.013 (-0.054, 0.028) |
| CCL11 | 0.0004 (-0.033, 0.034) | -0.012 (-0.039, 0.014) | -0.019 (-0.055, 0.016) | -0.0003 (-0.033, 0.033) | -0.005 (-0.031, 0.020) | -0.016 (-0.051, 0.019) | -0.024 (-0.057, 0.008) | -0.010 (-0.036, 0.016) | -0.008 (-0.042, 0.027) |
| CCL 2 | 0.002 (-0.027, 0.030) | 0.003 (-0.027, 0.033) | -0.001 (-0.040, 0.039) | -0.018 (-0.045, 0.009) | 0.002 (-0.028, 0.031) | -0.004 (-0.044, 0.035) | -0.007 (-0.035, 0.021) | 0.013 (-0.016, 0.042) | 0.019 (-0.019, 0.057) |
| CCL 3 | -0.007 (-0.050, 0.035) | -0.005 (-0.042, 0.032) | 0.001 (-0.035, 0.037) | -0.008 (-0.050, 0.033) | 0.006 (-0.031, 0.042) | 0.005 (-0.030, 0.041) | -0.041 (-0.084, 0.003) | -0.040 (-0.077, -0.003)* | -0.033 (-0.067, 0.001) |
| CCL 4 | -0.019 (-0.064, 0.026) | -0.007 (-0.070, 0.055) | -0.006 (-0.066, 0.055) | -0.029 (-0.072, 0.014) | 0.022 (-0.037, 0.081) | 0.007 (-0.050, 0.065) | -0.052 (-0.094, -0.009)* | -0.056 (-0.117, 0.005) | -0.029 (-0.088, 0.030) |
| CRP | -0.003 (-0.010, 0.005) | -0.014 (-0.029, 0.001) | -0.034 (-0.068, -0.0004)* | -0.003 (-0.011, 0.004) | -0.014 (-0.029, 0.001) | -0.032 (-0.065, 0.0003) | -0.006 (-0.013, 0.002) | -0.013 (-0.028, 0.002) | -0.024 (-0.056, 0.009) |
| CXCL10 | 0.00002 (-0.022, 0.023) | -0.004 (-0.032, 0.024) | -0.022 (-0.072, 0.027) | -0.003 (-0.025, 0.019) | -0.017 (-0.045, 0.012) | -0.025 (-0.073, 0.022) | -0.031 (-0.052, -0.010)** | -0.027 (-0.053, -0.0002)* | -0.044 (-0.087, -0.002)* |
| IFNγ | 0.0004 (-0.003, 0.004) | -0.003 (-0.009, 0.003) | -0.008 (-0.022, 0.007) | -0.001 (-0.005, 0.003) | -0.005 (-0.012, 0.001) | -0.019 (-0.032, -0.005)* | 0.002 (-0.002, 0.005) | 0.001 (-0.005, 0.006) | -0.006 (-0.020, 0.008) |
| IL-1β | -0.001 (-0.002, 0.001) | 0.0002 (-0.003, 0.003) | -0.003 (-0.009, 0.003) | -0.001 (-0.002, 0.001) | -0.001 (-0.004, 0.002) | -0.003 (-0.009, 0.003) | 0.0003 (-0.001, 0.002) | 0.001 (-0.002, 0.003) | -0.001 (-0.007, 0.005) |
| IL-10 | 0.001 (-0.0002, 0.003) | 0.001 (-0.001, 0.003) | 0.003 (-0.001, 0.007) | 0.001 (-0.001, 0.002) | 0.001 (-0.001, 0.003) | 0.003 (-0.001, 0.007) | -0.002 (-0.004, -0.001)** | -0.001 (-0.003, 0.001) | -0.002 (-0.006, 0.003) |
| IL-12p70 | 0.0003 (-0.0004, 0.0010) | -0.0001 (-0.001, 0.001) | 0.001 (-0.002, 0.003) | 0.0004 (-0.0003, 0.0011) | -0.0001 (-0.001, 0.001) | 0.002 (-0.0003, 0.004) | -0.0004 (-0.001, 0.0003) | -0.001 (-0.002, 0.0001) | -0.001 (-0.003, 0.002) |
| IL-6 | -0.003 (-0.007, 0.002) | -0.004 (-0.011, 0.003) | 0.001 (-0.015, 0.018) | -0.005 (-0.009, -0.0005)* | -0.008 (-0.015, -0.001)* | -0.002 (-0.018, 0.014) | -0.006 (-0.010, -0.002)** | -0.007 (-0.014, 0.0003) | -0.020 (-0.035, -0.005)* |
| IL-8 | -0.001 (-0.007, 0.004) | -0.0005 (-0.009, 0.008) | 0.004 (-0.011, 0.019) | 0.0003 (-0.005, 0.005) | -0.001 (-0.009, 0.007) | -0.002 (-0.018, 0.014) | -0.005 (-0.009, -0.0002)* | -0.003 (-0.011, 0.005) | 0.001 (-0.014, 0.016) |
| MPO | 0.005 (-0.010, 0.019) | 0.009 (-0.012, 0.030) | 0.004 (-0.031, 0.039) | 0.006 (-0.008, 0.020) | -0.007 (-0.028, 0.014) | -0.015 (-0.049, 0.020) | 0.003 (-0.011, 0.017) | -0.003 (-0.023, 0.017) | -0.003 (-0.037, 0.030) |
| TNFα | 0.001 (-0.003, 0.006) | -0.002 (-0.008, 0.003) | -0.005 (-0.015, 0.004) | 0.002 (-0.003, 0.006) | -0.003 (-0.008, 0.003) | -0.006 (-0.015, 0.004) | -0.006 (-0.010, -0.002)* | -0.004 (-0.009, 0.002) | -0.002 (-0.011, 0.008) |
| TNFRSF1B | 0.004 (-0.024, 0.032) | -0.006 (-0.035, 0.024) | -0.021 (-0.061, 0.019) | -0.012 (-0.039, 0.015) | -0.019 (-0.047, 0.010) | -0.020 (-0.060, 0.020) | -0.026 (-0.053, 0.001) | -0.047 (-0.076, -0.019)*** | -0.045 (-0.083, -0.007)* |
| Leukocytes | 0.0003 (-0.034, 0.034) | -0.038 (-0.070, -0.006)* | -0.009 (-0.052, 0.033) | 0.013 (-0.019, 0.045) | -0.016 (-0.049, 0.018) | -0.031 (-0.073, 0.012) | -0.026 (-0.058, 0.006) | -0.058 (-0.092, -0.024)*** | -0.073 (-0.116, -0.029)* |
| Vascular |  |  |  |  |  |  |  |  |  |
| EGF | 0.009 (-0.053, 0.070) | 0.009 (-0.064, 0.081) | -0.017 (-0.106, 0.071) | -0.038 (-0.099, 0.022) | -0.012 (-0.080, 0.057) | -0.048 (-0.128, 0.033) | 0.012 (-0.049, 0.073) | -0.013 (-0.082, 0.057) | -0.058 (-0.137, 0.021) |
| EphA2 | 0.001 (-0.026, 0.027) | 0.011 (-0.013, 0.035) | -0.006 (-0.032, 0.020) | -0.009 (-0.036, 0.017) | 0.006 (-0.019, 0.030) | -0.014 (-0.038, 0.010) | -0.026 (-0.053, 0.001) | -0.019 (-0.042, 0.005) | -0.015 (-0.039, 0.010) |
| E-selectin | -0.007 (-0.065, 0.052) | 0.028 (-0.039, 0.096) | 0.017 (-0.052, 0.085) | 0.002 (-0.059, 0.064) | 0.005 (-0.062, 0.073) | -0.004 (-0.074, 0.067) | -0.045 (-0.106, 0.015) | -0.084 (-0.148, -0.020)* | -0.072 (-0.139, -0.004)* |
| ICAM1 | -0.014 (-0.044, 0.015) | 0.005 (-0.023, 0.034) | -0.018 (-0.075, 0.040) | -0.022 (-0.052, 0.007) | -0.007 (-0.035, 0.021) | -0.041 (-0.096, 0.014) | -0.026 (-0.054, 0.002) | -0.025 (-0.053, 0.002) | -0.025 (-0.079, 0.030) |
| N-Cadherin | 0.018 (-0.003, 0.039) | 0.016 (-0.010, 0.041) | 0.004 (-0.037, 0.045) | -0.004 (-0.024, 0.016) | -0.003 (-0.028, 0.023) | -0.028 (-0.068, 0.013) | -0.012 (-0.032, 0.007) | -0.014 (-0.040, 0.011) | -0.020 (-0.061, 0.021) |
| P-selectin | -0.004 (-0.047, 0.039) | 0.005 (-0.032, 0.042) | -0.007 (-0.106, 0.093) | 0.003 (-0.040, 0.045) | -0.007 (-0.044, 0.030) | -0.036 (-0.132, 0.061) | -0.038 (-0.079, 0.002) | -0.046 (-0.081, -0.010)* | -0.045 (-0.142, 0.053) |
| VCAM1 | -0.019 (-0.060, 0.022) | -0.059 (-0.112, -0.005)* | -0.005 (-0.070, 0.060) | -0.011 (-0.051, 0.030) | -0.051 (-0.103, 0.001) | -0.029 (-0.094, 0.035) | -0.007 (-0.047, 0.033) | -0.052 (-0.103, -0.0003)* | -0.075 (-0.136, -0.015)* |
| VEGF | 0.008 (-0.040, 0.055) | -0.038 (-0.089, 0.014) | 0.011 (-0.069, 0.090) | -0.013 (-0.059, 0.034) | -0.056 (-0.106, -0.005)* | -0.052 (-0.125, 0.021) | -0.039 (-0.084, 0.006) | -0.046 (-0.095, 0.003) | -0.085 (-0.160, -0.009)* |
| Neurodegeneration |  |  |  |  |  |  |  |  |  |
| Aβ40 | 0.007 (-0.064, 0.079) | -0.012 (-0.064, 0.041) | -0.028 (-0.082, 0.026) | 0.035 (-0.035, 0.105) | -0.0005 (-0.051, 0.050) | -0.023 (-0.075, 0.029) | -0.049 (-0.117, 0.019) | -0.053 (-0.103, -0.002)* | -0.036 (-0.090, 0.018) |
| Aβ42 | 0.031 (-0.042, 0.103) | 0.029 (-0.024, 0.081) | 0.002 (-0.060, 0.064) | 0.044 (-0.025, 0.113) | 0.031 (-0.020, 0.081) | 0.046 (-0.015, 0.107) | -0.038 (-0.109, 0.033) | -0.046 (-0.096, 0.004) | -0.033 (-0.093, 0.028) |
| α-synuclein | 0.013 (-0.032, 0.058) | -0.008 (-0.057, 0.041) | -0.049 (-0.135, 0.037) | 0.025 (-0.021, 0.071) | 0.005 (-0.043, 0.054) | 0.050 (-0.035, 0.134) | -0.029 (-0.075, 0.017) | -0.044 (-0.091, 0.003) | 0.010 (-0.072, 0.092) |
| BDNF | 0.009 (-0.055, 0.073) | 0.029 (-0.030, 0.089) | 0.008 (-0.058, 0.074) | -0.030 (-0.091, 0.031) | 0.017 (-0.042, 0.075) | 0.003 (-0.062, 0.068) | 0.009 (-0.053, 0.071) | 0.028 (-0.030, 0.086) | 0.005 (-0.058, 0.067) |
| GFAP | -0.005 (-0.017, 0.007) | -0.017 (-0.029, -0.005)** | -0.028 (-0.052, -0.005)* | -0.001 (-0.012, 0.010) | -0.006 (-0.017, 0.006) | -0.025 (-0.049, -0.001)* | 0.004 (-0.007, 0.014) | -0.004 (-0.015, 0.008) | 0.002 (-0.023, 0.026) |
| NfL | -0.008 (-0.023, 0.007) | -0.005 (-0.023, 0.013) | 0.004 (-0.024, 0.031) | -0.003 (-0.017, 0.010) | -0.006 (-0.023, 0.011) | -0.007 (-0.035, 0.021) | -0.009 (-0.023, 0.005) | -0.010 (-0.027, 0.008) | -0.020 (-0.048, 0.007) |
| p-tau 181 | 0.015 (-0.012, 0.041) | 0.018 (-0.010, 0.046) | 0.012 (-0.028, 0.051) | 0.024 (-0.001, 0.049) | 0.009 (-0.018, 0.037) | -0.011 (-0.050, 0.028) | -0.023 (-0.048, 0.003) | -0.024 (-0.050, 0.002) | -0.036 (-0.073, 0.001) |
| p-tau 217 | 0.015 (-0.001, 0.031) | 0.004 (-0.017, 0.025) | 0.006 (-0.030, 0.042) | 0.012 (-0.004, 0.028) | 0.007 (-0.014, 0.028) | -0.004 (-0.039, 0.032) | -0.006 (-0.022, 0.009) | -0.008 (-0.029, 0.013) | -0.024 (-0.059, 0.011) |
| S100B | 0.008 (-0.054, 0.071) | -0.011 (-0.064, 0.041) | -0.013 (-0.093, 0.066) | -0.013 (-0.075, 0.049) | -0.036 (-0.087, 0.015) | -0.065 (-0.139, 0.008) | -0.070 (-0.128, -0.012)* | -0.039 (-0.093, 0.015) | -0.079 (-0.154, -0.005)* |
| t-tau | 0.046 (0.010, 0.083)* | 0.046 (0.001, 0.090)* | 0.004 (-0.051, 0.059) | 0.025 (-0.010, 0.061) | 0.032 (-0.011, 0.075) | 0.006 (-0.048, 0.060) | 0.003 (-0.032, 0.037) | 0.016 (-0.026, 0.058) | 0.005 (-0.048, 0.057) |

Beta coefficients are taken from separate quantile regression models (one for each dietary pattern-biomarker combination) adjusted for age, sex, education, occupation, living arrangement, physical activity, smoking status, energy intake, and fasting status. *p < 0.05; **q < 0.10, ***q<0.05 (FDR-adjusted using Benjamini–Hochberg); CI (confidence interval).

Aβ40 =amyloid beta protein 40; Aβ42 = amyloid beta protein 42; BDNF = Brain-derived neurotrophic factor; CCL11 = C-C motif chemokine 11; CCL2 = C-C motif chemokine 2; CCL3 = C-C motif chemokine 3; CCL4 = C-C motif chemokine 11; CRP = C-reactive protein; CXCL10 = chemokine CXC ligand 10; EGF = Epidermal growth factor; EphA2 = ephrin type-A receptor 2; GDF15 = growth differentiation factor 15; GFAP = Glial fibrillary acidic protein; γGT = gamma-glutamyl transferase; HbA1c = hemoglobin A1c; ICAM1 = Intercellular Adhesion Molecule 1; IFNγ = Interferon gamma; IGFBP1 = Insulin-like growth factor binding protein 1; IL-1b = interleukin 1beta; IL-10 = interleukin 10; IL-12p70 = Interleukin 12p70; IL-6 = interleukin 6; IL-8 = interleukin 8; MPO = Myeloperoxidase; NfL = neurofilament light chain; p-tau181 = phosphorylated tau 181; p-tau 217 = phosphorylated tau 217; S100B = S100 calcium-binding protein B; T4 = Thyroxine; TNF α= tumor necrosis factor alpha; TNFRSF1B = tumor necrosis factor receptor superfamily member 1B; t-tau = Total tau; VCAM1 = vascular cell adhesion protein 1; VEGF = vascular endothelial growth factor

**Table S8**: Mediation of the significant associations between dietary pattern adherence (per 1-SD) and blood-based biomarkers by body mass index.

| Dietary pattern | Biomarker | Indirect effect  β (95% CI) | Direct effect  β (95% CI) | Total effect  β (95% CI) | Proportion mediated (95% CI) |
| --- | --- | --- | --- | --- | --- |
| AMED | FA | 0.000 (-0.001, 0.004) | 0.034 (-0.016, 0.084) | 0.035 (-0.014, 0.085) | 0.041 (-0.061, 0.144) |
|  | GFAP | -0.002 (-0.005, 0.001) | -0.015 (-0.041, 0.011) | -0.017 (-0.043, 0.009) | 0.110 (-0.121, 0.342) |
| AHEI | FA | 0.000 (-0.002, 0.001) | 0.009 (-0.035, 0.053) | 0.009 (-0.036, 0.053) | -0.053 (-0.425, 0.317) |
|  | GDF15 | 0.000 (-0.002, 0.001) | -0.084 (-0.129, -0.039) * | -0.085 (-0.129, -0.040) * | 0.004 (-0.015, 0.024) |
| rEDII | WBC | -0.002 (-0.006, 0.002) | -0.050 (-0.082, -0.019) * | -0.052 (-0.083, -0.021) * | 0.034 (-0.047, 0.115) |
|  | ALP | -0.003 (-0.008, 0.001) | -0.085 (-0.138, -0.032) * | -0.089 (-0.141, -0.036) * | 0.039 (-0.014, 0.093) |
|  | TNFRSF1B | -0.003 (-0.007, 0.001) | -0.056 (-0.095, -0.017) * | -0.059 (-0.098, -0.021) * | 0.052 (-0.027, 0.130) |
|  | Insulin | -0.014 (-0.026, -0.002) * | -0.082 (-0.127, -0.038) * | -0.096 (-0.143, -0.050) * | 0.146 (0.023, 0.269) * |
|  | LEP | -0.037 (-0.068, -0.006) * | -0.058 (-0.091, -0.025) * | -0.095 (-0.140, -0.050) * | 0.389 (0.147, 0.631) * |
|  | GDF15 | -0.002 (-0.005, 0.001) | -0.104 (-0.147, -0.060) * | -0.105 (-0.149, -0.062) * | 0.0167 (-0.014, 0.048) |

Beta coefficients represent natural indirect, natural direct, and total effects estimated using causal mediation analysis. Negative coefficients indicate that higher dietary pattern adherence is associated with lower biomarker levels, while positive coefficients indicate the opposite. Only associations significant after multiple comparison correction (Benjamini-Hochberg threshold: q < 0.1) in the main analyses were included. Models were adjusted for age, sex, education, occupation, living status, tobacco use, physical activity, fasting status, and total energy intake. The mediator (body mass index, continuous) and blood-biomarker outcomes were modelled using linear regression. Proportion mediated represents the percentage of the total effect of dietary pattern adherence on the blood-biomarker that is explained by body mass index. A positive proportion indicates that the mediator explains part of the association between dietary pattern adherence and the biomarker, while a negative proportion indicates that the indirect and direct effects operate in opposite directions. * p<0.05 for indirect, direct, and total effects and proportion mediated.

After excluding participants with missing data on body mass index, the final analytical sample size was 1754.

AMED: Alternative Mediterranean Diet
AHEI: Alternative Healthy Eating Index
rEDII: Reversed Empirical Dietary Inflammatory Score

FA = folic acid; GFAP = glial fibrillary acidic protein; GDF15 = growth differentiation factor 15; WBC = leukocytes; ALP = alkaline phosphatase;
LEP = leptin; TNFRSF1B = tumour necrosis factor receptor superfamily member 1B

**Table S9**: Mediation of the significant associations between dietary pattern adherence (per 1-SD) and blood-based biomarkers by number of chronic diseases.

| Dietary pattern | Biomarker | Indirect effect  β (95% CI) | Direct effect  β (95% CI) | Total effect  β (95% CI) | Proportion mediated  (95% CI) |
| --- | --- | --- | --- | --- | --- |
| AMED | FA | 0.001 (-0.003, 0.005) | 0.036 (-0.012, 0.085) | 0.037 (-0.012, 0.087) | 0.018 (-0.078, 0.115) |
|  | GFAP | 0.001 (-0.001, 0.002) | -0.019 (-0.045, 0.007) | -0.018 (-0.044, 0.007) | -0.016 (-0.113, 0.080) |
| AHEI | FA | -0.001 (-0.005, 0.003) | 0.013 (-0.031, 0.057) | 0.012 (-0.032, 0.056) | -0.091 (-0.570, 0.385) |
|  | GDF15 | -0.002 (-0.011, 0.006) | -0.086 (-0.129, -0.042 * | -0.088 (-0.133, -0.043) * | 0.029 (-0.065, 0.124) |
| rEDII | WBC | -0.006 (-0.011, -0.002) * | -0.046 (-0.077, -0.015) * | -0.052 (-0.083, -0.021) * | 0.126 (0.014, 0.237) * |
|  | ALP | -0.005 (-0.009, -0.001) * | -0.077 (-0.131, -0.023) * | -0.082 (-0.136, -0.029) * | 0.057 (-0.013, 0.127) |
|  | TNFRSF1B | -0.006 (-0.010, -0.002) * | -0.052 (-0.089, -0.014) * | -0.058 (-0.096, -0.020) * | 0.113 (0.017, 0.208) * |
|  | Insulin | -0.015 (-0.024, -0.006) * | -0.080 (-0.126, -0.035) * | -0.095 (-0.141, -0.049) * | 0.160 (0.046, 0.274) * |
|  | LEP | -0.020 (-0.030, -0.009) * | -0.078 (-0.120, -0.035) * | -0.098 (-0.141, -0.055) * | 0.205 (0.078, 0.331) * |
|  | GDF15 | -0.014 (-0.022, -0.006) * | -0.096 (-0.138, -0.054) * | -0.110 (-0.154, -0.067) * | 0.132 (0.051, 0.212) * |

Beta coefficients represent natural indirect, natural direct, and total effects estimated using causal mediation analysis. Negative coefficients indicate that higher dietary pattern adherence is associated with lower biomarker levels, while positive coefficients indicate the opposite. Only associations significant after multiple comparison correction (Benjamini-Hochberg threshold: q < 0.1) in the main analyses were included. Models were adjusted for age, sex, education, occupation, living status, tobacco use, physical activity, fasting status, and total energy intake. The mediator (chronic disease burden, count) was modelled using Poisson regression and blood-biomarker outcomes using linear regression. Proportion mediated represents the percentage of the total effect of dietary pattern adherence on the blood-biomarker that is explained by chronic disease count. A positive proportion indicates that the mediator explains part of the association between dietary pattern adherence and the biomarker, while a negative proportion indicates that the indirect and direct effects operate in opposite directions. * p<0.05 for indirect, direct, and total effects and proportion mediated.

AMED: Alternative Mediterranean Diet
AHEI: Alternative Healthy Eating Index
rEDII: Reversed Empirical Dietary Inflammatory Score

FA = folic acid; GFAP = glial fibrillary acidic protein; GDF15 = growth differentiation factor 15; WBC =leukocytes; ALP = alkaline phosphatase; LEP = leptin; TNFRSF1B = tumour necrosis factor receptor superfamily member 1B

**Figure S1:** Flowchart of study participants

*Blood sample not collected includes technical difficulties during blood collection (N=91) and refusal of blood sampling for health check or research (N=164). Research blood unavailable, insufficient, or unusable includes refusal of research blood donation (N=171), insufficient blood volume for research storage (N=132), and unusable blood samples for biomarker analysis (N=276).

**Additional details on biomarker missingness are provided in Supplementary Table 6.

**Figure S2:** Directed acyclic graph (DAG) describing the potential causal and mediating effects of diet quality on blood-based biomarkers.

Green arrows represent a causal path between dietary pattern adherence (exposure; green oval: ►), potential mediators (blue ovals), and blood-based biomarkers (outcome; blue oval: ▌). Pink arrows indicate potential confounding pathways where potential confounding factors (orange ovals) may influence the associations between the exposure and outcome.

**Figure S3:** Volcano plots displaying the association between dietary pattern adherence (per 1-SD increment) and blood-based biomarkers at the 25^th^ percentile of the biomarkers’ distributions.

Beta coefficients are taken from separate quantile regression models (one for each dietary pattern-biomarker combination) adjusted for age, sex, education, occupation, living arrangement, physical activity, smoking status, fasting status, and energy intake. The absolute value of each coefficient represents the strength of the association between adherence to a given dietary pattern and levels of a blood-based biomarker (across different pathophysiological domains), while the sign indicates the direction (direct or inverse) of the relationship. The horizontal dashed grey line indicates the threshold for statistical significance (p < 0.05). The horizontal dashed red line indicates the Benjamini-Hochberg corrected threshold for statistical significance (q < 0.10) and the solid red line indicates a more stringent threshold (q < 0.05).

AMED: Alternative Mediterranean Diet
AHEI: Alternative Healthy Eating Index

rEDII: Reversed Empirical Dietary Inflammatory Score

CysC = Cystatin C; CXCL10 = C-X-C motif chemokine ligand 10; FA = Folic acid; GGT = Gamma-glutamyl transferase; IL-6 = Interleukin 6; IL-10 = Interleukin 10

**Figure S4:** Volcano plots displaying the association between dietary pattern adherence (per 1-SD increment) and blood-based biomarkers at the 75^th^ percentile of the biomarkers’ distributions.

Beta coefficients are taken from separate quantile regression models (one for each dietary pattern-biomarker combination) adjusted for age, sex, education, occupation, living arrangement, physical activity, smoking status, fasting status, and energy intake. The absolute value of each coefficient represents the strength of the association between adherence to a given dietary pattern and levels of a blood-based biomarker (across different pathophysiological domains), while the sign indicates the direction (direct or inverse) of the relationship. The horizontal dashed grey line indicates the threshold for statistical significance (p < 0.05). The horizontal solid red line indicates the Bonferroni-corrected threshold (p < 0.05/162). No associations met the Benjamini–Hochberg thresholds for statistical significance (q < 0.10 or q < 0.05).

AMED: Alternative Mediterranean Diet
AHEI: Alternative Healthy Eating Index

rEDII: Reversed Empirical Dietary Inflammatory Score

**Figure S5:** Volcano plots displaying associations between dietary pattern adherence (per 1-SD increment) at wave 1 and routinely collected blood-biomarkers at the median (50th percentile) of their distributions across waves 1 and 3.

Beta coefficients are taken from separate quantile regression models (one for each dietary pattern-biomarker combination) adjusted for age, sex, education, occupation, living arrangement, physical activity, smoking status, fasting status, and energy intake. The absolute value of each coefficient represents the strength of the association between adherence to a given dietary pattern and levels of a blood-based biomarker (across different pathophysiological domains), while the sign indicates the direction (direct or inverse) of the relationship The horizontal dashed grey line indicates the threshold for statistical significance (p < 0.05). The horizontal dashed red line indicates the Benjamini-Hochberg corrected threshold for statistical significance (q < 0.10). The horizontal solid red line indicates a stricter Benjamini-Hochberg threshold (q < 0.05) at wave 1 and a Bonferroni-corrected threshold (p < 0.05/39) at wave 3. No associations met the Benjamini–Hochberg thresholds for statistical significance (q < 0.10 or q < 0.05) at wave 3.

After excluding participants missing any biomarker data at wave 1 and wave 3, the final analytical sample size was 1368.

AMED: Alternative Mediterranean Diet
AHEI: Alternative Healthy Eating Index

rEDII: Reversed Empirical Dietary Inflammatory Score

ALB = Albumin, ALP = Alkaline Phosphatase, Ca = Calcium, Cr = Creatinine, FA = Folic acid, GGT = Gamma glutamyl transferase, Hb = Haemoglobin, HbA1c = Glycated haemoglobin, TSH = Thyroid stimulating hormone, T4 = Thyroxine, TC = Total cholesterol, VitB12 = Vitamin B12, WBC = Leukocytes

**Figure S6:** Volcano plots displaying the association between dietary pattern adherence (per 1-SD increment) and blood-based biomarkers at the 50^th^ percentile of the biomarkers’ distributions, among participants with complete data on all potential confounders

Beta coefficients are taken from separate quantile regression models (one for each dietary pattern-biomarker combination) adjusted for age, sex, education, occupation, living arrangement, physical activity, smoking status, fasting status, and energy intake. The absolute value of each coefficient represents the strength of the association between adherence to a given dietary pattern and levels of a blood-based biomarker (across different pathophysiological domains), while the sign indicates the direction (direct or inverse) of the relationship. The horizontal dashed grey line indicates the threshold for statistical significance (p < 0.05). The horizontal dashed red line indicates the Benjamini-Hochberg corrected threshold for statistical significance (q < 0.10) and the solid red line indicates a more stringent threshold (q < 0.05).

After excluding participants with missing data on potential confounders, the final analytical sample size was 1619.

AMED: Alternative Mediterranean Diet
AHEI: Alternative Healthy Eating Index

rEDII: Reversed Empirical Dietary Inflammatory Score

ALP = Alkaline phosphatase; FA = Folic acid; GDF 15 = Growth differentiation factor 15; GFAP = Glial fibrillary acidic protein; LEP = Leptin; TNFRSF1B = Tumor necrosis factor receptor superfamily member 1B; WBC = Leukocytes.

**Figure S7**: Volcano plots displaying associations between dietary pattern adherence (per 1-SD increment) and blood-based biomarkers at the median (50th percentile) of their distributions among participants with four or fewer chronic diseases at baseline.

Beta coefficients are taken from separate quantile regression models for participants with ≤ 4 chronic diseases (one for each dietary pattern-biomarker combination) adjusted for age, sex, education, occupation, living arrangement, physical activity, smoking status, fasting status, and energy intake. The absolute value of each coefficient represents the strength of the association between adherence to a given dietary pattern and levels of a blood-based biomarker (across different pathophysiological domains), while the sign indicates the direction (direct or inverse) of the relationship The horizontal dashed grey line indicates the threshold for statistical significance (p<0.05). The horizontal dashed red line indicates the Benjamini-Hochberg corrected threshold for statistical significance (q<0.10), and the solid red line indicates a more stringent threshold (q < 0.05).

After excluding participants with > 4 chronic diseases, the final analytical sample size was 1239.

AMED: Alternative Mediterranean Diet
AHEI: Alternative Healthy Eating Index

rEDII: Reversed Empirical Dietary Inflammatory Score

Ab40 = Amyloid beta 40; ALP = Alkaline phosphatase; B2M = Beta‑2 microglobulin; CCL4 = C‑C motif chemokine ligand 4; Cpep = C‑peptide; CXCL10 = C‑X‑C motif chemokine ligand 10; FA = Folic acid; GDF15 = Growth differentiation factor 15; GFAP = Glial fibrillary acidic protein; GGT = Gamma‑glutamyltransferase; LEP = Leptin; TC = Total cholesterol; TNFRSF1B = Tumour necrosis factor receptor superfamily member 1B; VitB12 = Vitamin B12; WBC = Leukocytes

**Figure S8**: Volcano plots displaying associations between dietary pattern adherence (per 1-SD increment) and blood-based biomarkers at the median (50th percentile) of their distributions, additionally adjusted for number of medications.

Beta coefficients are taken from separate quantile regression models (one for each dietary pattern-biomarker combination) adjusted for age, sex, education, occupation, living arrangement, physical activity, smoking status, fasting status, energy intake and number of medications. The absolute value of each coefficient represents the strength of the association between adherence to a given dietary pattern and levels of a blood-based biomarker (across different pathophysiological domains), while the sign indicates the direction (direct or inverse) of the relationship The horizontal dashed grey line indicates the threshold for statistical significance (p<0.05). The horizontal dashed red line indicates the Benjamini-Hochberg corrected threshold for statistical significance (q<0.10), and the solid red line indicates a more stringent threshold (q < 0.05).

AMED: Alternative Mediterranean Diet
AHEI: Alternative Healthy Eating Index

rEDII: Reversed Empirical Dietary Inflammatory Score

ALP = Alkaline phosphatase; CysC = Cystatin C; FA = Folic acid; GFAP = Glial fibrillary acidic protein; GDF 15 = Growth differentiation factor 15; LEP = Leptin; TNFRSF1B = Tumor necrosis factor receptor superfamily member 1B; WBC = Leukocytes.

**Figure S9:** Volcano plots displaying the association between dietary pattern adherence (per 1-SD increment) and blood-based biomarkers at the median (50^th^ percentile) of the biomarkers’ distributions, additionally adjusted for chronic disease count.

Beta coefficients are taken from separate quantile regression models (one for each dietary pattern-biomarker combination) adjusted for age, sex, education, occupation, living arrangement, physical activity, smoking status, energy intake, and chronic disease count. The absolute value of each coefficient represents the strength of the association between adherence to a given dietary pattern and levels of a blood-based biomarker (across different pathophysiological domains), while the sign indicates the direction (direct or inverse) of the relationship. The horizontal dashed line indicates the threshold for statistical significance (p < 0.05). The horizontal solid line indicates the Benjamini-Hochberg corrected threshold for statistical significance (q < 0.10).

AMED: Alternative Mediterranean Diet
AHEI: Alternative Healthy Eating Index
rEDII: Reversed Empirical Dietary Inflammatory Score

Ab40 = Amyloid beta 40; Adipo = Adiponectin; ALP = Alkaline phosphatase; CCL4 = C C motif chemokine ligand 4; CRP = C-reactive protein; CysC = Cystatin C; ESel = E-selectin; FA = Folic acid; GDF15 = Growth differentiation factor 15; GFAP = Glial fibrillary acidic protein; Hb = Haemoglobin; IFNG = Interferon gamma; LEP = Leptin; PSel = P-selectin; TNFRSF1B = Tumour necrosis factor receptor superfamily member 1B; TSH = Thyroid-stimulating hormone; VCAM1 = Vascular cell adhesion molecule 1; VitB12 = Vitamin B12; WBC = Leukocytes.

**Figure S10:** Volcano plots displaying the association between dietary pattern adherence (per 1-SD increment) and blood-based biomarkers at the median (50^th^ percentile) of the biomarkers’ distributions, weighted using inverse probability weighting.

Beta coefficients are taken from separate quantile regression models (one for each dietary pattern-biomarker combination) adjusted for age, sex, education, occupation, living arrangement, physical activity, smoking status, energy intake and chronic disease count. The absolute value of each coefficient represents the strength of the association between adherence to a given dietary pattern and levels of a blood-based biomarker (across different pathophysiological domains), while the sign indicates the direction (direct or inverse) of the relationship. The horizontal dashed line indicates the threshold for statistical significance (p < 0.05). The horizontal solid line indicates the Benjamini-Hochberg corrected threshold for statistical significance (q < 0.10).

Quantile regression models used stabilized inverse probability weights to account for potential selection into the analytic sample. Weights were derived from a logistic regression model predicting inclusion (versus exclusion due to missing biomarker, dietary, or covariate data) from age, sex, education, occupation, living arrangement, smoking status, physical activity, fasting status, total energy intake, chronic disease burden, number of medications, and BMI.

AMED: Alternative Mediterranean Diet
AHEI: Alternative Healthy Eating Index
rEDII: Reversed Empirical Dietary Inflammatory Score

ALP = Alkaline phosphatase; B2M = Beta 2 microglobulin; CCL3 = C C motif chemokine ligand 3; Cpep = C peptide; CXCL10 = C X C motif chemokine ligand 10; CysC = Cystatin C; FA = Folic acid; GDF15 = Growth differentiation factor 15; GFAP = Glial fibrillary acidic protein; GGT = Gamma glutamyl transferase; LEP = Leptin; PSel = P-selectin; TNFRSF1B = Tumour necrosis factor receptor superfamily member 1B; ttau = Total tau; VitB12 = Vitamin B12; WBC =Leukocytes..

**Figure S11:** Volcano plots displaying sex-specific associations between dietary pattern adherence (per 1-SD increment) and blood-based biomarkers at the median (50th percentile) of their distributions

The only interaction between diet and sex that was statistically significant was that for associations between AHEI and CXC motif chemokine ligand 10 (p for interaction = 0.001) after false discovery rate correction.

Beta coefficients are taken from separate quantile regression models (one for each dietary pattern-biomarker combination) adjusted for age, education, occupation, living arrangement, physical activity, smoking status, fasting status, energy intake, and a multiplicative interaction term between the dietary pattern and sex. The absolute value of each coefficient represents the strength of the association between adherence to a given dietary pattern and levels of a blood-based biomarker (across different pathophysiological domains), while the sign indicates the direction (direct or inverse) of the relationship. The horizontal dashed grey line indicates the threshold for statistical significance (p < 0.05). The horizontal dashed red line indicates the Benjamini-Hochberg corrected threshold for statistical significance (q < 0.10), and the solid red line indicates a more stringent threshold (q < 0.05).

AMED: Alternative Mediterranean Diet
AHEI: Alternative Healthy Eating Index
rEDII: Reversed Empirical Dietary Inflammatory Score

ALP = Alkaline phosphatase; FA = Folic acid; GFAP = Glial fibrillary acidic protein; GDF-15 = Growth differentiation factor 15; IL-6 = Interleukin-6; LEP = Leptin; ttau = Total tau

**Figure S12:** Volcano plots displaying the unadjusted association between dietary pattern adherence (per 1-SD increment) and blood-based biomarkers at the median (50^th^ percentile) of the biomarkers’ distributions.

Beta coefficients are taken from separate unadjusted quantile regression models (one for each dietary pattern-biomarker combination). The absolute value of each coefficient represents the strength of the association between adherence to a given dietary pattern and levels of a blood-based biomarker (across different pathophysiological domains), while the sign indicates the direction (direct or inverse) of the relationship. The horizontal dashed line indicates the threshold for statistical significance (p < 0.05). The horizontal solid line indicates the Benjamini-Hochberg corrected threshold for statistical significance (q < 0.10).

AMED: Alternative Mediterranean Diet
AHEI: Alternative Healthy Eating Index
rEDII: Reversed Empirical Dietary Inflammatory Score

Ab40 = Amyloid beta 40; ALP = Alkaline phosphatase; B2M = Beta 2 microglobulin; Cpep = C peptide; Cr = Creatinine; CRP = C-reactive protein; CXCL10 = C X C motif chemokine ligand 10; CysC = Cystatin C; FA = Folic acid; GDF15 = Growth differentiation factor 15; GFAP = Glial fibrillary acidic protein; GGT = Gamma glutamyltransferase; ICAM1 = Intercellular adhesion molecule 1; IGFBP1 = Insulin-like growth factor binding protein 1; IL-1b = Interleukin 1 beta; IL-6 = Interleukin 6; LEP = Leptin; NCAD = N-cadherin; NFL = Neurofilament light chain; ptau181 = Phosphorylated tau 181; ptau217 = Phosphorylated tau 217; TNFa = Tumour necrosis factor alpha; TNFRSF1B = Tumour necrosis factor receptor superfamily member 1B; VCAM1 = Vascular cell adhesion molecule 1; VitB12 = Vitamin B12; WBC = Leukocytes.
